# Genomic‐based epidemiology reveals independent origins and gene flow of glyphosate resistance in *Bassia scoparia* populations across North America

**DOI:** 10.1111/mec.16215

**Published:** 2021-10-21

**Authors:** Karl Ravet, Crystal D. Sparks, Andrea L. Dixon, Anita Küpper, Eric P. Westra, Dean J. Pettinga, Patrick J. Tranel, Joel Felix, Don W. Morishita, Prashant Jha, Andrew Kniss, Phillip W. Stahlman, Paul Neve, Eric L. Patterson, Philip Westra, Todd A. Gaines

**Affiliations:** ^1^ Department of Agricultural Biology Colorado State University Fort Collins Colorado USA; ^2^ Rothamsted Research West Common Harpenden Hertfordshire UK; ^3^ Center for Outcomes Research and Epidemiology College of Veterinary Medicine Kansas State University Manhattan Kansas USA; ^4^ Crop Science Division Weed Control Bayer AG Frankfurt am Main Germany; ^5^ Department of Crop Sciences University of Illinois Urbana Illinois USA; ^6^ Oregon State University, Malheur Experiment Station Ontario OR USA; ^7^ Kimberly Research and Extension Center University of Idaho Kimberly Idaho USA; ^8^ Department of Agronomy Iowa State University Ames Iowa USA; ^9^ Department of Plant Sciences University of Wyoming Laramie Wyoming USA; ^10^ Kansas State University Agricultural Research Center Hays Kansas USA; ^11^ Department of Plant & Environmental Sciences University of Copenhagen Taastrup Denmark; ^12^ Department of Plant, Soil, and Microbial Sciences Michigan State University East Lansing Michigan USA; ^13^ Present address: Department of Soil and Crop Sciences Colorado State University Fort Collins Colorado USA

**Keywords:** gene duplication, gene flow, herbicide resistance, independent evolution, mobile genetic elements, population genetics

## Abstract

Genomic‐based epidemiology can provide insight into the origins and spread of herbicide resistance mechanisms in weeds. We used kochia (*Bassia scoparia*) populations resistant to the herbicide glyphosate from across western North America to test the alternative hypotheses that (i) a single *EPSPS* gene duplication event occurred initially in the Central Great Plains and then subsequently spread to all other geographical areas now exhibiting glyphosate‐resistant kochia populations or that (ii) gene duplication occurred multiple times in independent events in a case of parallel evolution. We used qPCR markers previously developed for measuring the structure of the *EPSPS* tandem duplication to investigate whether all glyphosate‐resistant individuals had the same *EPSPS* repeat structure. We also investigated population structure using simple sequence repeat markers to determine the relatedness of kochia populations from across the Central Great Plains, Northern Plains and the Pacific Northwest. We found that the original *EPSPS* duplication genotype was predominant in the Central Great Plains where glyphosate resistance was first reported. We identified two additional *EPSPS* duplication genotypes, one having geographical associations with the Northern Plains and the other with the Pacific Northwest. The *EPSPS* duplication genotype from the Pacific Northwest seems likely to represent a second, independent evolutionary origin of a resistance allele. We found evidence of gene flow across populations and a general lack of population structure. The results support at least two independent evolutionary origins of glyphosate resistance in kochia, followed by substantial and mostly geographically localized gene flow to spread the resistance alleles into diverse genetic backgrounds.

## 1 INTRODUCTION

The rate at which herbicide resistance evolves in plant populations is dictated by the interplay of genetic and ecological factors (Hawkins et al., 2019; Kreiner et al., 2018), which determine whether resistance emerges and spreads via (i) a small number of initial evolutionary origins, or even a single evolutionary origin, followed by subsequent dispersal across landscapes; or (ii) via numerous local, independent evolutionary origins with limited and localized dispersal (Baucom, 2019). Important genetic determinants include the potential for resistance to evolve from standing genetic variation versus the requirement for populations to “wait” for de novo variation to arise via mutation after the onset of selection (Barrett & Schluter, 2008), which in turn dictates the likelihood of soft versus hard selective sweeps for herbicide resistance in plant populations (Hermisson & Pennings, 2005; Messer & Petrov, 2013). In the absence of standing variation, the time for novel adaptive mutations to arise depends on the size of the mutational target (the number of adaptive mutations/genotypes that endow resistance) and fitness trade‐offs (Neve et al., 2014; Vila‐Aiub et al., 2009). Ultimately, these genetic factors determine the extent to which the emergence of resistance is mutation‐limited.

If molecular genetics establishes the rate at which adaptive variation arises, ecological factors determine the rate at which newly evolved or pre‐adapted genotypes disperse between populations and across landscapes. In highly mobile and migratory organisms, such as migratory insects, relatively infrequent mutations may be dispersed long distances, whereas in less mobile species (including plants), resistance spread is likely to be more limited, making it relatively more likely that widely distributed resistance traits arise from multiple independent mutational origins (Beckie et al., 2021). However, in agroecosystems, the dispersal of arthropods, weeds and pathogens may often be facilitated by human activities. This is particularly true for weed seeds that are often moved across landscapes by farm machinery and potentially across continents in contaminated crop seed (Benvenuti, 2007), meaning that pollen‐ and/or seed‐mediated dispersal is more frequent than would be predicted by consideration of ecological factors (Beckie et al., 2019).

Although these genetic and ecological factors are fundamentally important for understanding the rate of adaptation of plant populations to herbicides and for tailoring appropriate resistance management guidelines, relatively few studies have explored regional‐scale patterns of resistance frequency and dispersal or associated genetic and genomic signatures of resistance alleles. In Europe, studies focused on landscape‐, national‐ and continental‐level patterns of resistance to acetyl CoA carboxylase (ACCase‐) and acetolactate synthase (ALS‐) inhibiting herbicides have implicated multiple, independent evolutionary origins of herbicide resistance alleles in weeds (Délye et al., 2010; Dixon et al., 2021; Menchari et al., 2006). Multiple origins of resistance for the ACCase‐ and ALS‐inhibiting herbicide groups may be due in part to standing genetic variation for resistance to these herbicides (Délye et al., 2013), multiple possibilities for target‐site mutations (Gaines et al., 2020) and the lack of large fitness costs for resistance to these herbicides (Vila‐Aiub et al., 2009). In North America, studies focusing on the evolution and spread of glyphosate resistance have been more equivocal, with some support for multiple independent origins (Okada et al., 2013), some studies presenting evidence for both long‐distance dispersal and local evolution (Kreiner et al., 2019), and others implicating a single evolutionary origin with subsequent widespread dispersal (Molin, Patterson, et al., 2020; Molin et al., 2018).

Here we use a combination of molecular population genetics and genomic‐based epidemiology (defined here as the use of genomic data to determine the origin(s) and spread of known resistance genotypes) to review evidence for the evolutionary origins of glyphosate resistance within and between multiple populations of the agricultural weed kochia [*Bassia scoparia* (L.) A. J. Scott, synonymous with *Kochia scoparia* (L.) Schrad.], an introduced weed that occurs in the semi‐arid arable lands of the western USA and Canada (Friesen et al., 2009). Kochia populations from North America exhibit high levels of genetic diversity but a lack of strong population structure (Kumar et al., 2019; Martin et al., 2020; Mengistu & Messersmith, 2002), probably due to several reproductive traits that promote cross‐pollination and long‐range dispersal of pollen and seed (Beckie et al., 2016).

Genome sequencing has revealed that the structural rearrangement that caused duplication of the gene encoding the glyphosate target enzyme (5‐enolpyruvylshikimate‐3‐phosphate synthase, *EPSPS*) in glyphosate‐resistant kochia is due to complex interactions between mobile genetic elements and local tandem rearrangements (Jugulam et al., 2014; Patterson et al., 2019). These molecular mechanisms are unlikely to spontaneously generate the same genotype in multiple kochia populations and thus we are presented with a unique opportunity to employ genomic‐based epidemiology to address questions about the evolutionary origins of glyphosate resistance in kochia. If many individuals from different populations contain the same structural rearrangement, this would provide strong evidence for a single evolutionary origin. If this is true, analysing patterns of neutral genetic variation using more traditional population genetics approaches will identify evidence of relatedness and gene flow between glyphosate‐resistant populations. If populations exist that lack this exact rearrangement and instead contain an *EPSPS* gene duplication with a different structural rearrangement, this would provide evidence for an independent evolutionary origin. Alternatively, an *EPSPS* gene duplication with a different structural rearrangement could provide evidence for a shared evolutionary origin followed by a subsequent rearrangement.

## 2 MATERIALS AND METHODS

### 2.1 Plant materials

Seeds were collected from kochia individuals from locations in the western USA and western Canada between 2010 and 2015 (Table 1). Crops grown at the sampled locations included winter wheat, no‐till fallow and sugar beet. A total of 44 kochia populations from eight different states in the USA and one province in Canada were used for the analyses (Table 1). A population is defined as the seed from kochia individuals isolated from a single field at a geographically distinct location. The number of individuals sampled per population varied from five to 20, with at least 100 seeds sampled per plant and collecting from plants across at least 0.5 ha. Glyphosate‐susceptible (GS) populations were collected from several locations in Kansas, Colorado and Oregon. Populations suspected to contain glyphosate‐resistant (GR) individuals were collected throughout Kansas, Colorado, Oregon, Idaho, Oklahoma, Texas, Montana and Alberta (Table 1). Populations from Colorado (Westra et al., 2019), Oregon and Idaho (Kumar et al., 2018), and Montana (Kumar et al., 2014) were previously screened for glyphosate resistance.

**TABLE 1:** List of kochia (*Bassia scoparia*) populations used in the SSR study of population genetics

Population	Country	State/Province	City or County	Year	*n* (plant)	Resistance
CO1R	USA	Colorado	Akron^a^	2012	18	R
CO2R	USA	Colorado	Brush	2012	18	R
CO3R	USA	Colorado	Cope	2012	18	R
CO4R	USA	Colorado	Julesburg	2011	18	R
CO5R	USA	Colorado	Kit Carson	2013	18	R
CO6R	USA	Colorado	Otis	2012	18	R
CO7R	USA	Colorado	Strasburg	2012	18	R
CO8R	USA	Colorado	Strasburg	2014	18	R
ID1R	USA	Idaho	Ada	2014	9	R
ID2R	USA	Idaho	Ada	2014	9	R
KS1S	USA	Kansas	Barton	2012	9	S^b^
KS2S	USA	Kansas	Finney	2012	9	S^b^
KS3R	USA	Kansas	Gray	2012	9	R
KS4R	USA	Kansas	Greeley	2012	9	R
KS5S	USA	Kansas	Meade	2012	9	S^b^
KS6S	USA	Kansas	Ness	2012	9	S^b^
KS7S	USA	Kansas	Philip	2012	9	S
KS8S	USA	Kansas	Pratt	2012	9	S^b^
KS9R	USA	Kansas	Scott	2012	9	R
KS10R	USA	Kansas	Scott	2012	9	R
KS11R	USA	Kansas	Stevens	2012	9	R
KS12R	USA	Kansas	Thomas	2012	9	R
KS13R	USA	Kansas	Thomas	2007	9	R
KS14R	USA	Kansas	Wallace	2012	9	R
KS15R	USA	Kansas	Wichita	2012	9	R
MT1R	USA	Montana	Chester	2012	9	R
MT2R	USA	Montana	Gilford	2012	9	R
MT3R	USA	Montana	Joplin	2012	9	R
OK1R	USA	Oklahoma	Cimarron	2012	9	R
OR1R	USA	Oregon	Malheur	2014	9	R
OR2R	USA	Oregon	Malheur	2014	9	R
OR3R	USA	Oregon	Malheur	2014	9	R
OR4R	USA	Oregon	Malheur	2014	9	R
OR5R	USA	Oregon	Malheur	2014	9	R
OR6R	USA	Oregon	Malheur	2014	9	R
OR7R	USA	Oregon	Malheur	2014	9	R
OR9S	USA	Oregon	Malheur	2015	9	S
TX1R	USA	Texas	Hartley		12	R
TX2R	USA	Texas	Hartley		18	R
TX3R	USA	Texas	Hartley		18	R
TX4R	USA	Texas	Hartley		18	R
TX5R	USA	Texas	Hartley		18	R
WY1R	USA	Wyoming	Powell	2015	9	R
AB1R	CANADA	Alberta			18	R

Note

Name, origin and year of sampling for 44 populations. *n* corresponds to the number of plants (individuals) sampled for leaf tissue from each population. Glyphosate resistance (R) corresponds to populations with at least one individual surviving treatment with glyphosate at 840 g a.e. ha^−1^. Populations with no survival were considered susceptible (S).

^a^ Population M32, glyphosate‐resistant line used to sequence *EPSPS* duplication region (Patterson et al., 2019; Westra et al., 2019).

^b^ Population suspected to be R when collected but all tested individuals had S phenotype; population could be heterogeneous with R at low frequency.

### 2.2 Glyphosate resistance screening

A population ID was assigned to each locality (Table 1). Each population ID consists of the abbreviated state/province of origin, a unique identifying number, and its designation as GR or GS (e.g., CO1R = Colorado resistant population 1). To determine variation in each population for glyphosate susceptibility and resistance, a screening was performed in the glasshouse. Seeds from each population were planted in germination flats. After emergence, seedlings were transplanted into 18‐insert (7 × 7‐cm pots) flats containing custom mix potting soil (Fafard, Sun Gro Horticulture), and grown at 23°C under a 14‐hr light/10‐hr dark cycle with supplemental light from sodium halide lamps. Plants were watered daily and fertilized once (Miracle‐Gro, Scotts Miracle‐Gro Company). When plants were 3 weeks old, a leaf was collected for DNA extraction (see below) and nine to 18 plants per population (Table 1) were sprayed with commercially formulated glyphosate (Roundup WeatherMax) in distilled water at 0.84 kg acid equivalent ha^−1^. Glyphosate applications were performed using a moving flat‐fan nozzle (8002EVS) in a laboratory spray chamber at 156 L ha^−1^ spray volume. Three weeks after herbicide treatment, individual survival for each population was assessed. Populations for which at least one individual survived were classified as GR.

### 2.3 DNA extraction and genotyping

For total genomic DNA extraction, leaf tissue was collected from plants grown as previously described prior to glyphosate treatment. Samples were immediately frozen in liquid nitrogen and stored at −80°C. Tissue was ground using a TissueLyser II (Qiagen; 30 Hz for 2 min). Genomic DNA was extracted from 100 mg fresh weight tissue following a modified cetyltrimethylammonium bromide (CTAB) extraction protocol (Doyle, 1991). DNA quality and concentration were measured using a NanoDrop spectrophotometer (Thermo Scientific ND‐1000). All DNA samples were normalized to 5 ng µl^−1^ with deionized water.

### 2.4 Genomic‐based epidemiology using *EPSPS* copy number and associated duplication markers

The *EPSPS* locus has been sequenced from a single GR kochia individual using BAC libraries (Patterson et al., 2019). The *EPSPS* repeat unit was variable with two units being most common: (i) a full‐length repeat called type I containing *EPSPS* and six other flanking genes and (ii) a less frequent form called type II containing *EPSPS* and only three other flanking genes. A ~15‐kb mobile genetic element (MGE) was present both upstream and downstream of the entire tandem duplication. Additionally, a copy of the MGE was found between every repeat unit, indicating the MGE has been coduplicated after subsequent crossing‐over events (Patterson et al., 2019). Once the structure of the repeat was determined, quantitative polymerase chain reaction (qPCR) markers that specifically amplify the two types of repeats and the MGE were developed to confirm the sequence and measure the copy number of each part of the repeat structure. Genomic DNA from 113 individuals representing 27 of the populations evaluated for glyphosate resistance above, along with 36 individuals representing 11 populations used in Gaines et al. (2016) and 58 individuals representing 18 populations collected in Montana, was used for real‐time PCR to measure the relative copy number of genomic *EPSPS*, as well as the longer type I (56.1 kb) and shorter type II (32.7 kb) segments associated with *EPSPS* duplication, and the MGE Fhy3/FAR1 (Patterson et al., 2019). Primer sequences for these features as well as the normalization gene *Acetolactate Synthase* (*ALS*) were (i) *EPSPS*, For (5′‐CGCTATATGTTGGATGCTCTAAG‐3′), Rev (5′‐CACTCCTATTCTCTTTACCAGC‐3′); (ii) Type I (56.1 kb), For (5′‐GACGGAAATACCCTCAATATAGACA‐3′), Rev (5′‐ACGCCCAAGATGTACATTGATA‐3′); (iii) Type II (32.7 kb), For (5′‐GACGGAAATACCCTCAATATAGACA‐3′), Rev (5′‐CATGCCTTTGATGTCCAAGTTT‐3′); (iv) MGE, For (5′‐GAAGATAGCGAGACGTTTGAG‐3′), Rev (5′‐CGGCTTGATCGGTTAAGATAC‐3′); and (v) *ALS*, For (5′‐CCAGAAAAGGCTGCGATG‐3′), Rev (5′‐CTGACTCGCTCTGATTCCA‐3′). A GR control (population M32 in Patterson et al., 2019, COR1 in this study) with high *EPSPS* copy number, presence of both type I and type II *EPSPS* duplication segments, and an increased MGE copy number was included along with a susceptible control (7710) containing a single copy of *EPSPS*, no copies of type I or II markers, and a low copy number of the MGE. The qPCR protocol of Patterson et al. (2019) was used and relative copy number was calculated using the ∆Ct method (Schmittgen & Livak, 2008). The type I and type II *EPSPS* duplication qPCR primers have a forward primer in the MGE and a reverse primer in either the type I or the type II sequence, respectively, enabling amplification only when the MGE is located next to the type I or type II repeat segment. Populations and their genotypes and geographical locations were plotted in R using the ggplot and sf packages (R Core Team, 2019).

### 2.5 SSR genotyping

To develop polymorphic genetic markers for kochia, Roche 454 sequencing technology (Keck Center, University of Illinois) was used to determine partial genomic sequence from a single GR kochia individual taken from the KS‐R1 population used in Wiersma et al. (2015). Approximately 75.2 million aligned bases (from 357 million total bases sequenced) with reads having an average length of 557 bases were obtained. This data set was screened for simple sequence repeats (SSRs) using Imperfect SSR Finder (https://data.nal.usda.gov/dataset/imperfect‐ssr‐finder). Primers to amplify the identified SSRs were also designed using Imperfect SSR Finder ffollowing the approach described by Lee et al. (2009). Primers were evaluated to amplify SSRs with pentanucleotide repeat units to use as molecular markers for genotyping. Out of a total of 30 SSR markers initially tested, 11 (Table 2) exhibiting polymorphisms were selected for genotyping the 44 kochia populations.

**TABLE 2:** List of SSR primers used for genotyping

SSR name	Forward primer sequence (5′–3′)	*T*_m_	Reverse primer sequence (5′–3′)	*T*_m_	Amplicon size (bp)	Motif repeat	Annealing temperature (°C)
162	TGATGTGAAAAGAACACCCC	58.4	TGTGATTCCAGGGAGGAGTA	58.1	216	(ATTTG)n	62
1225	GGTCCCAATGACAAACAGTC	57.8	GTTGGGTTTGGTTCTTGTTG	58.0	183	(CCCAA)n	62
1792	AACTAGTCGGATCGAGCCTT	58.0	AATCACACAACTCCGCAAGT	58.2	174	(CCCAA)n	57
2656	AACCAAACCGCACTAAACTG	57.8	GCACAATAGAGAGGGCAAAA	58.0	277	(TGGTT)n	62
2895	GTCATAGCCATCCCTTACCC	58.3	TATTGCCCTGTTCTTCAGGA	58.3	267	(AGTTC)n	62
2916	GTGCCAAAACCAAAGTTGTC	58.1	CCTCTCAACACAGGTTGCTT	57.9	215	(ATTTT)n	62
3332	CATGTACCTCGTGCAATGAA	58.1	TTTAGCTTAGCAATCACGGG	58.1	203	(TGTTG)n	57
5417	AGTGTGCTAAGAATTTGGGC	57.0	ACCATCAATTGTGATCGGAG	58.4	203	(GATAT)n	62
5608	GAGGCAAAGGATAAGGTGGT	58.1	ACGAAGGGAAGAGAAAGGAA	58.0	249	(AGGGA)n	57
5726	GCAGCCAAGCCATTCTATTA	58.0	AGCCCTTCCATGGAGAATTT	59.9	223	(TTATT)n	62
8376	ATGGAGCTGAACTGAACCAA	58.3	TTGTACCAGAATGCCTGTCA	57.7	254	(CTGAA)n	62

Note

For each locus, primer sequence and melting temperature (*T*_m_) and targeted repeat motif are provided. PCR expected amplicon size and annealing temperature used for PCR are also indicated.

Amplification of 100‐ to 200‐bp sequence regions containing the selected SSR markers was carried out using PCR with specific primers (Table 2; together with expected fragment size of the amplified loci). Amplification of 5 ng of genomic DNA was performed using EconoTaq PLUS Master Mix (Lucigen) in a BioRad CFX96 Real‐Time System (C1000 Touch Thermal Cycler). After an initial denaturation period of 2 min at 94°C, PCR was run for 37 cycles, consisting of denaturation at 94°C for 30 s, annealing at either 57°C or 62°C for 30 s (Table 2), and extension at 72°C for 45 s. A final extension of 2 min at 72°C was included. Amplified fragment size analysis was carried out by capillary electrophoresis using an Agilent Fragment Analyzer, using the 35–500‐bp dsDNA method. Fragments were sized by prosize 2.0 software. Alleles were binned using flexibin software (Amos, 2005; Amos et al., 2007).

### 2.6 Genetic diversity and population structure

The evaluation of linkage equilibrium and Hardy–Weinberg equilibrium of loci was done using exact tests with the functions test_LD and test_HW, respectively in the “genepop” package (version 1.1.7; Rousset, 2008). Descriptive summaries for each population across loci were calculated using the divBasic function in the “diveRsity” package (version 1.9.90; Keenan et al., 2013). Descriptive summaries for each locus across populations were calculated using the locus_table function in the “poppr” package (version 2.8.6; Kamvar et al., 2014, 2015).

Missing data were assessed using the info_table function in the “poppr” package (Kamvar et al., 2014, 2015) and loci with more than 10% data missing and individuals with more than 20% data missing were removed. Descriptive summaries for populations and loci were made on the entire data set and again on the data set after removing loci and individuals with unacceptable levels of missing data. A population‐level phylogeny based on the neighbour‐joining clustering method using Prevosti's genetic distance model was generated with bootstrapped support using 1000 replicates with the aboot function in the “poppr” package (Kamvar et al., 2014, 2015) and plotted using the Interactive Tree of Life version 4 (Letunic & Bork, 2019). A principal component analysis (PCA) was performed using the “adegenet” (version 2.1.3; Jombart & Ahmed, 2011) and “ade4” (version 1.7‐16; Dray & Dufour, 2007) packages.

Model‐based putative population clustering was performed using structure version 2.3.4 (Pritchard et al., 2000). The number of genetic groups (*K*) present within the 509 individuals tested was determined running a continuous series of *K* = 1–22. The program was run with a burn‐in of 30,000 and a run‐length of 100,000 Markov chain Monte Carlo (MCMC) replications in 20 independent runs using the LOCPRIOR model (sampling location information included) to account for weak structure signals in the data set. The most likely number of clusters was determined using the Evanno method (Evanno et al., 2005) as implemented in structure harvester version 0.6.94 (Earl & vonHoldt, 2012). The final analysis for *K* = 3 was performed using a burn‐in of 50,000 and 500,000 MCMC replications with 20 independent runs. Runs were summarized using clumpp version 1.1.2b (Jakobsson & Rosenberg, 2007) utilizing the Greedy algorithm and visualized with distruct version 1.1 (Rosenberg, 2004).

## 3 RESULTS

### 3.1 Genomic‐based epidemiology using *EPSPS* copy number and associated duplication markers

Glyphosate resistance in kochia was first detected in Kansas in 2007, followed by Colorado and Alberta in 2012, Oklahoma and Montana in 2013, and Texas, Wyoming, Idaho and Oregon in 2014 (Heap, 2020). We surveyed a set of populations from across western North America for glyphosate resistance (Table 1). Phenotypic variation for glyphosate resistance was observed in most populations. Most populations returned the anticipated phenotype for resistance or susceptibility based on agronomic experience of the collector; however, a few populations were designated as suspected GR based on agronomic field observations while all nine individuals tested had a GS phenotype in our screening assay, resulting in a classification for the population as GS but with potential for it to be heterogeneous for phenotype (containing GR at a low frequency). Most populations were therefore not completely susceptible or completely resistant, but rather contained a quantifiable proportion of each as continuous variation. For example, both KS5S and KS6S were suspected to be GR when sampled from the field but were classified as GS by phenotyping (Table 1) and had GR individuals based on *EPSPS* gene copy number data (Table S1).

**FIGURE 1:**
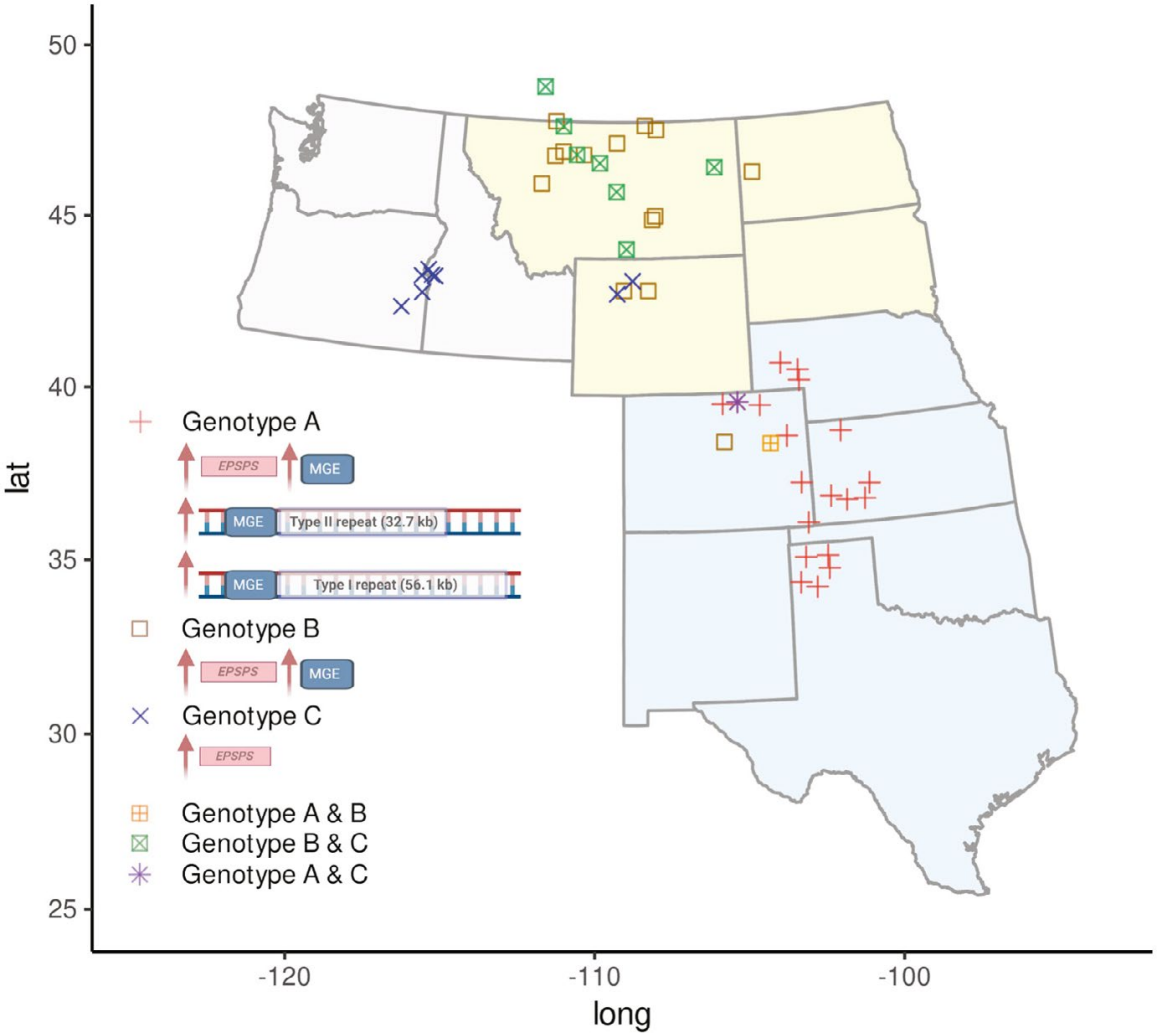
Map of three *EPSPS* genotypes in glyphosate‐resistant kochia (*Bassia scoparia*). Genotype A, increased *EPSPS* copy number, presence of type I and II repeats, MGE ≥ 10; Genotype B, increased *EPSPS* copy number, no type I or II, MGE ≥ 10; Genotype C, increased *EPSPS* copy number, no type I or II, MGE < 10. Geographical regions are Central Great Plains (light blue), Northern Great Plains (light yellow) and Pacific Northwest (light grey). Some populations contained multiple genotypes (see Table 2 for population‐level genotype proportions). Figure key created using biorender

**FIGURE 2:**
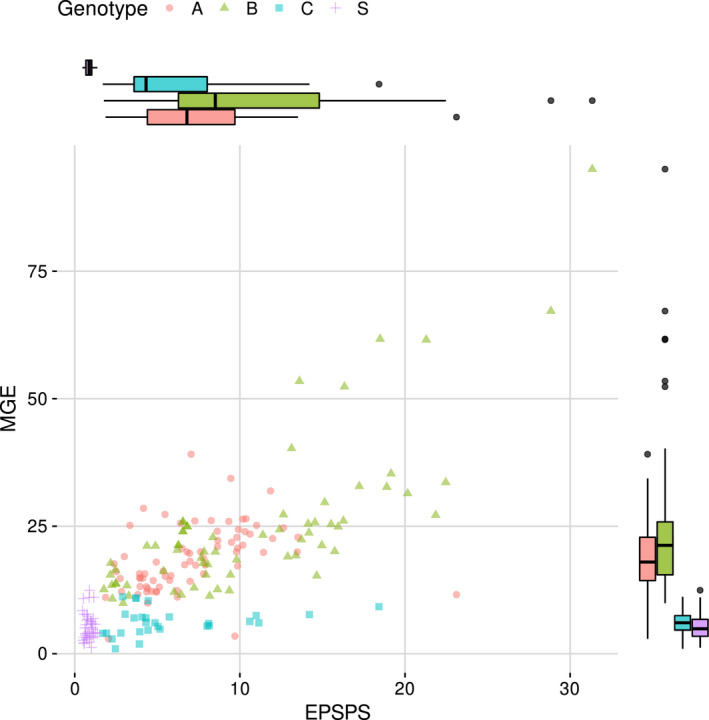
Relationship of EPSPS copy number and MGE copy number in glyphosate‐resistant kochia (*Bassia scoparia*). Genotype A defined as increased *EPSPS* copy number, presence of type I and II repeats, MGE ≥ 10; Genotype B defined as increased *EPSPS* copy number, no type I or II repeats, MGE ≥ 10; Genotype C defined as increased *EPSPS* copy number, no type I or II, MGE < 10; Genotype S defined as *EPSPS* copy number of 1. Box and whisker plots represent mean, lower quartile and upper quartile for each genotype for MGE or *EPSPS* copy number

Using four PCR markers, we quantified the number of copies per haploid genome for *EPSPS*, type I repeat (56.1 kb), type II repeat (32.7 kb), and the MGE in individuals from multiple populations. We defined three genotypes based on these four qPCR markers, using *EPSPS* > 1.4 to define increased *EPSPS* above the wild‐type *EPSPS* gene copy number of 1.0 per haploid genome; type I and type II > 0 to define presence of the two markers of *EPSPS* gene duplication described by Patterson et al. (2019); and MGE < 10 defined as “normal” (within the range of variation observed in most GS plants) and ≥10 defined as “increased” MGE. The three categories of *EPSPS*‐duplication genotypes, were defined as follows: (A) increased *EPSPS*, type I and II repeats, and MGE copy numbers that correspond to increased *EPSPS* copy number (≥10) (Central Great Plains); (B) increased *EPSPS*, no type I or II repeats, MGE ≥ 10 (Northern Plains); and (C) increased *EPSPS*, no type I or II repeats, MGE < 10 (Pacific Northwest, north‐central Wyoming) (Figure 1). Although all GR individuals from across the continent had increased *EPSPS* copy number, kochia in the Pacific Northwest and Northern Plains did not contain the type I and II repeats associated with Genotype A identified in the tandem *EPSPS* duplication in the previously characterized population from Colorado (Figure 1, Table 3). In contrast, the type I and II repeats were present in the Central Great Plains, and in ratios consistent with those reported by Patterson et al. (2019) (Table S1). Genotype A was only found in the Central Great Plains, with Genotype B dominant in the Northern Plains and Genotype C most common in the Pacific Northwest. The proportion of each genotype in each population is shown in Table 3. In the Central Great Plains, Genotype A was consistent with the initial characterization of the *EPSPS* repeat structure (Patterson et al., 2019) in that type I and II PCR markers always amplified together, and type I (long repeat) was nearly always present at higher copy number than type II (short repeat) (Table 2; Table S1). Both Genotype A and Genotype B showed a positive correlation between *EPSPS* copy number and MGE copy number (Figure 2), while Genotype C had no increase in MGE with increasing *EPSPS* copy number (Figure 2). With few exceptions, individuals with wild‐type *EPSPS* copy number of 1 (S genotypes) had no increase in MGE copy number (Figure 2; Table S1). Some individuals had much higher *EPSPS* copy number than previously reported; for example, individuals collected in Montana had 20–30 copies with no presence of type I or II and very high (>60 copies) MGE (Table 3; Table S1).

**TABLE 3:** Population means and standard errors for *EPSPS*, type I repeat, type II repeat, and mobile genetic element motif (MGE) along with proportion of genotypes A, B, C and S in all kochia (*Bassia scoparia*) populations, grouped by geographical region

Geographical region	Population	Location	*n*	*EPSPS* mean	*EPSPS SE*	Type I mean	Type I *SE*	Type II mean	Type II *SE*	MGE mean	MGE *SE*	Proportion of genotype			
												A	B	C	S
Pacific Northwest	ID1R	Idaho	4	4.7	2.0	0.0	—	0.0	—	4.9	0.5	0.0	0.0	0.5	0.5
	OR1R‐5R	Oregon	5	3.6	0.4	0.0	—	0.0	—	5.2	1.2	0.0	0.0	1.0	0.0
Central Great Plains	CO1R	Colorado	5	10.3	0.8	11.9	0.8	3.9	0.5	22.5	1.4	1.0	0.0	0.0	0.0
	CO2R	Colorado	3	0.8	0.1	0.0	—	0.0	—	3.5	1.6	0.0	0.0	0.0	1.0
	CO3R	Colorado	9	2.6	0.4	0.9	0.7	0.4	0.2	13.8	1.4	0.2	0.6	0.0	0.2
	CO4R	Colorado	3	8.7	0.6	10.8	0.7	3.2	0.2	20.9	1.6	1.0	0.0	0.0	0.0
	CO5R	Colorado	3	6.9	1.6	10.1	2.2	3.0	0.7	24.5	6.2	1.0	0.0	0.0	0.0
	CO6R	Colorado	3	1.1	0.1	0.0	—	0.0	—	6.3	0.3	0.0	0.0	0.0	1.0
	CO9S	Colorado	3	1.0	0.0	0.0	—	0.0	—	56.0	0.1	0.0	0.0	0.0	1.0
	KS12R	Kansas	6	6.4	1.3	8.1	1.7	2.6	0.5	17.1	3.4	1.0	0.0	0.0	0.0
	KS3R	Kansas	6	7.0	0.7	8.2	1.0	2.4	0.4	16.6	0.8	1.0	0.0	0.0	0.0
	KS4R	Kansas	7	5.1	3.1	5.9	3.1	2.6	1.6	10.4	1.7	0.6	0.0	0.0	0.4
	KS5S	Kansas	5	2.0	0.9	1.4	1.4	0.4	0.4	6.1	2.3	0.2	0.0	0.0	0.8
	KS6S	Kansas	8	4.8	0.9	9.2	1.3	3.0	0.4	20.4	2.2	1.0	0.0	0.0	0.0
	KS9R	Kansas	2	5.9	3.8	5.9	3.8	3.8	0.2	3.2	0.3	1.0	0.0	0.0	0.0
	OK1R	Oklahoma	7	5.9	1.6	7.4	2.3	2.4	0.8	15.2	3.3	0.7	0.0	0.0	0.3
	SBK‐11	Colorado	3	8.3	0.8	11.6	0.6	3.1	0.0	20.9	0.5	1.0	0.0	0.0	0.0
	SBK‐14	Nebraska	2	4.3	0.1	5.5	0.0	1.7	0.0	12.4	2.4	1.0	0.0	0.0	0.0
	SBK‐14	Nebraska	1	0.7	—	0.0	—	0.0	—	3.6	—	0.0	0.0	0.0	1.0
	SBK‐20	Nebraska	3	5.8	2.7	6.4	3.6	2.1	1.2	16.6	4.9	0.7	0.0	0.0	0.3
	SBK‐22	Colorado	9	3.4	0.7	3.8	1.8	1.2	0.6	15.3	3.7	0.4	0.0	0.3	0.2
	SBK‐9	Colorado	6	1.9	0.3	0.0	—	0.0	—	10.3	1.9	0.0	0.7	0.0	0.3
	TX1R	Texas	2	8.9	1.0	9.0	3.4	3.0	0.8	20.9	3.5	1.0	0.0	0.0	0.0
	TX2R	Texas	3	7.6	0.9	9.3	0.5	3.8	0.4	21.6	2.3	1.0	0.0	0.0	0.0
	TX3R	Texas	3	5.3	0.8	4.6	0.1	1.7	0.1	13.2	0.6	1.0	0.0	0.0	0.0
	TX4R	Texas	4	9.7	2.0	9.6	2.3	3.4	0.8	19.7	2.1	1.0	0.0	0.0	0.0
	TX5R	Texas	5	8.3	2.0	8.6	1.6	3.7	0.7	18.2	3.1	1.0	0.0	0.0	0.0
Northern Plains	AB1R	Canada	14	5.9	0.8	0.0	—	0.0	—	16.6	2.5	0.0	0.6	0.3	0.1
	Beaverton	Montana	2	18.8	3.7	0.0	—	0.0	—	31.6	2.0	0.0	1.0	0.0	0.0
	Billings	Montana	10	15.4	0.6	0.0	—	0.0	—	25.8	1.7	0.0	1.0	0.0	0.0
	C_8	Montana	6	9.4	0.8	0.0	—	0.0	—	20.6	1.7	0.0	1.0	0.0	0.0
	C4	Montana	1	6.5	—	0.0	—	0.0	—	11.6	—	0.0	1.0	0.0	0.0
	Carter	Montana	8	5.6	1.3	0.0	—	0.0	—	10.3	2.2	0.0	0.6	0.4	0.0
	Charlis	Montana	2	15.6	6.2	0.0	—	0.0	—	21.3	5.9	0.0	1.0	0.0	0.0
	Cropland	Montana	2	30.1	1.3	0.0	—	0.0	—	81.1	13.9	0.0	1.0	0.0	0.0
	Cut Bank	Montana	3	5.6	1.5	0.0	—	0.0	—	15.6	4.4	0.0	1.0	0.0	0.0
	Denton	Montana	3	4.9	1.7	0.0	—	0.0	—	14.3	5.2	0.0	0.7	0.3	0.0
	GIL	Montana	8	11.1	1.6	0.0	—	0.0	—	15.8	2.5	0.0	0.75	0.25	0.0
	Greg	Montana	3	13.2	4.5	0.0	—	0.0	—	35.4	9.1	0.0	1.0	0.0	0.0
	Haven	Montana	4	16.6	2.0	0.0	—	0.0	—	54.2	5.0	0.0	1.0	0.0	0.0
	SBK‐29	Montana	2	0.8	0.1	0.0	—	0.0	—	9.9	2.5	0.0	0.0	0.0	1.0
	SBK‐31	Wyoming	6	3.5	0.9	0.0	—	0.0	—	4.8	0.6	0.0	0.0	0.7	0.3
	SBK‐41	Montana	2	0.7	0.1	0.0	—	0.0	—	4.5	0.5	0.0	0.0	0.0	1.0
	SBK‐42	Montana	2	0.7	0.0	0.0	—	0.0	—	4.8	0.9	0.0	0.0	0.0	1.0
	Teton	Montana	1	20.2	—	0.0	—	0.0	—	31.4	—	0.0	1.0	0.0	0.0
	Vida	Montana	2	16.3	2.1	0.0	—	0.0	—	8.4	0.8	0.0	0.0	1.0	0.0
	Wild	Montana	3	7.8	1.1	0.0	—	0.0	—	16.4	2.2	0.0	0.0	1.0	0.0
	WY1R	Wyoming	3	6.8	0.9	0.0	—	0.0	—	7.7	2.6	0.0	0.3	0.7	0.0

Note

Genotype A is defined as increased *EPSPS* copy number, presence of type I and II repeats, MGE ≥ 10; Genotype B is defined as increased *EPSPS* copy number, no type I or II repeats, and MGE ≥ 10; Genotype C is defined as increased *EPSPS* copy number, no type I or II repeats, MGE < 10; Genotype S is defined as *EPSPS* copy number of 1. Proportion colour scale from 0 (yellow) to 1 (green).

All GS kochia samples in the survey had one copy of *EPSPS* per haploid genome, no amplification of type I or II markers, and most had four to six copies of the MGE per haploid genome (Figure 2; Table S1). MGE copy number was increased to >10 in one GS individual from each of four populations (Figure 2; Table S1; located in Colorado, Nebraska, and Montana) without a corresponding increase in *EPSPS* gene duplication (Table S1).

### 3.2 Genetic diversity and population structure

To determine if the population genetics data supported the three independent origins identified by genomic‐based epidemiology, we developed 11 SSR markers (Table 2) and genotyped kochia populations collected from the three geographical regions to measure population‐level genetic diversity and genetic similarity of populations between localities. Across all populations, using Fisher's combined probability test, all SSR loci were in linkage equilibrium (*p* > .05), but not in Hardy–Weinberg equilibrium (HWE; *p* < .05). For descriptive summaries and the neighbour‐joining tree, the marker “SSR162” was removed as well as five individuals: KS2S_4, KS2S_5, KS2S_7, KS2S_8 and KS8S_2, as this locus had more than 10% missing data and the individuals had more than 20% missing data after “SSR162” was removed (Tables S2 and S3).

Allele counts, expected heterozygosity and evenness for all loci across all populations and then after the removal of individuals with missing data are reported in Table S2. Descriptive summaries of 44 populations at 10 SSR loci are presented in Table 4 (data for all 11 SSR loci are presented in Table S3). Populations ranged in their percentage of total alleles observed and allelic richness from 57.7% and 1.42 (KS13R) to 24.7% and 2.61 (CO7R). *F*_IS_ ranged from −0.04 (95% confidence interval [CI] = −0.28 to 0.13; KS13R) to 0.58 (95% CI = 0.33–0.79; MT3R). The *F*_IS_ results should be interpreted with caution noting that loci did not meet the assumptions of HWE (Waples, 2015) and many CIs spanned from negative to positive and over very large ranges.

**TABLE 4:** Descriptive summaries of 44 populations of kochia (*Bassia scoparia*) genotyped at 10 SSR loci

Population	Number of individuals genotyped	Percentage missing (%)	Number of alleles observed	Percentage of total alleles observed (%)	Allelic richness	*H*_O_	*H*_E_	*F*_IS_ (95% CI)
AB1R	17	4.71	32	48.95	2.22	0.26	0.44	0.39 (0.25–0.53)
CO1R	18	3.33	31	45.39	2.15	0.22	0.40	0.45 (0.35–0.56)
CO2R	18	2.78	34	50.24	2.52	0.41	0.48	0.16 (0.02–0.29)
CO3R	18	1.67	30	47.53	2.01	0.27	0.32	0.14 (−0.04 to 0.32)
CO4R	18	4.44	38	54.94	2.55	0.32	0.50	0.36 (0.23–0.48)
CO5R	18	6.11	31	46.02	2.04	0.30	0.37	0.18 (0.02–0.32)
CO6R	18	1.11	32	48.03	2.11	0.33	0.37	0.12 (−0.03 to 0.25)
CO7R	18	1.67	38	57.73	2.61	0.44	0.51	0.14 (0.00–0.26)
CO8R	18	2.78	32	49.18	2.30	0.41	0.46	0.11 (−0.02 to 0.23)
ID1R	8	0	28	45.61	2.30	0.29	0.42	0.31 (0.07–0.49)
ID2R	7	0	20	33.27	1.71	0.17	0.27	0.37 (−0.04 to 0.76)
KS10R	9	0	29	40.27	2.38	0.46	0.45	0.00 (−0.26 to 0.20)
KS11R	9	1.11	32	49.10	2.34	0.33	0.44	0.25 (0.09–0.35)
KS12R	9	0	29	43.13	2.37	0.38	0.43	0.12 (−0.07 to 0.27)
KS13R	9	2.22	17	24.74	1.42	0.14	0.13	−0.04 (−0.28 to 0.13)
KS14R	9	2.22	30	42.90	2.21	0.25	0.38	0.35 (0.13–0.52)
KS15R	9	5.56	33	48.42	2.51	0.36	0.46	0.22 (0.00–0.40)
KS1S	9	1.11	31	47.92	2.44	0.42	0.49	0.14 (−0.08 to 0.32)
KS2S^a^	5	18.00	18	29.02	1.56	0.18	0.25	0.29 (−0.06 to 0.54)
KS3R	9	0	29	44.46	2.35	0.31	0.51	0.39 (0.17–0.56)
KS4R	9	4.44	23	33.93	1.98	0.34	0.37	0.06 (−0.23 to 0.30)
KS5S	9	4.44	28	42.70	2.34	0.34	0.45	0.26 (0.06–0.40)
KS6S	9	4.44	32	48.45	2.42	0.33	0.47	0.29 (0.04–0.51)
KS7S	9	1.11	26	38.61	2.16	0.32	0.44	0.25 (−0.04 to 0.46)
KS8S^a^	8	8.75	23	33.61	1.91	0.20	0.36	0.45 (0.14–0.75)
KS9R	9	4.44	30	43.88	2.37	0.33	0.46	0.29 (0.02–0.48)
MT1R	9	2.22	20	31.46	1.60	0.18	0.19	0.05 (−0.24 to 0.33)
MT2R	9	5.56	26	39.50	2.09	0.31	0.39	0.20 (0.04–0.30)
MT3R	9	0	27	39.65	2.11	0.16	0.37	0.58 (0.33–0.79)
OK1R	9	3.33	24	36.87	2.00	0.24	0.37	0.33 (0.18–0.48)
OR1R	9	0	21	34.87	1.82	0.18	0.39	0.54 (0.21–0.80)
OR2R	9	0	36	40.06	2.04	0.21	0.34	0.37 (0.18–0.50)
OR3R	9	4.44	25	36.91	1.96	0.20	0.31	0.37 (0.10–0.56)
OR4R	9	2.22	19	31.88	1.58	0.14	0.20	0.29 (−0.30 to 0.61)
OR5R	9	3.33	20	32.68	1.68	0.20	0.28	0.29 (0.03–0.46)
OR6R	9	1.11	26	39.87	1.99	0.20	0.32	0.37 (0.16–0.51)
OR7R	9	0	25	41.55	2.03	0.20	0.40	0.50 (0.25–0.75)
OR9S	8	1.25	25	40.44	2.16	0.31	0.41	0.26 (0.06–0.42)
TX1R	12	0.83	34	46.33	2.43	0.33	0.41	0.21 (0.00–0.40)
TX2R	18	3.33	31	45.45	2.14	0.27	0.37	0.27 (0.11–0.43)
TX3R	18	1.67	30	46.70	2.16	0.31	0.39	0.21 (0.07–0.32)
TX4R	18	1.67	34	50.86	2.28	0.32	0.44	0.27 (0.12–0.40)
TX5R	16	2.50	31	46.67	2.14	0.40	0.41	0.01 (−0.16 to 0.19)
WY1R	9	3.33	32	46.16	2.56	0.36	0.52	0.31 (0.11–0.47)

Note

Number of individuals genotyped per population, percentage of missing data averaged across loci, number of alleles observed summed across all loci, percentage of the total alleles (*n* = 70) observed in each population averaged across loci, allelic richness averaged across all loci; *H*_O_, observed heterozygosity; *H*_E_, expected heterozygosity; and F_IS_ and 95% confidence interval (CI).

^a^ Populations KS2S (*n* = 4) and KS8S (*n* = 1) had individuals removed due to data missing > 20%.

**FIGURE 3:**
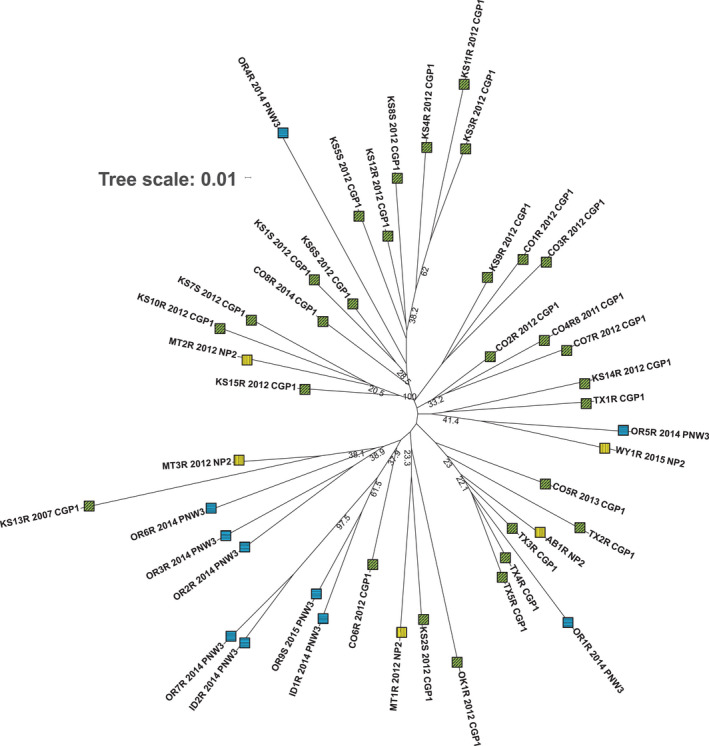
Population‐level phylogeny tree in glyphosate‐resistant kochia (*Bassia scoparia*). Forty‐four kochia populations are included in this analysis. The neighbour‐joining tree is based on Prevosti's distance. Bootstrap values (%) are shown if >20% and are based on 1000 replicates. Glyphosate‐resistant (R) and ‐susceptible (S); CGP1, Central Great Plains (green square with diagonal lines); NP2, Northern Plains (yellow square with vertical lines); PNW3, Pacific Northwest (blue square with horizontal lines)

**FIGURE 4:**
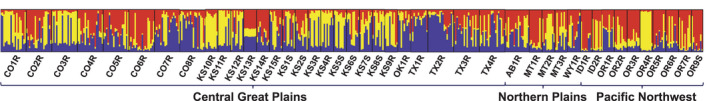
Bayesian clustering analysis (structure) in glyphosate‐resistant kochia (*Bassia scoparia*). Assignment of 509 kochia individuals from 44 populations to the *K* = 3 genetic clusters inferred by analysis. Populations are sorted by region (Central Great Plains, Northern Plains and Pacific Northwest) and alphabetized within region. Each horizontal bar corresponds to a distinct individual and its probability of assignment, *q*, to each cluster

As loci and populations did not meet the assumptions of HWE, a neighbour‐joining tree was used to assess genetic similarity between populations. This tree showed some expected groups by region, with 12 Central Great Plains populations grouped in a large clade supported by 100% bootstrap values (Figure 3). This clade also contained OR4R (Pacific Northwest) and MT2R (Northern Plains). The populations from the Pacific Northwest largely clustered together (OR2R, OR3R, OR6R, OR7R, OR9S, ID1R and ID2R) with the clade of populations OR9S and ID1R and OR7R and ID2R supported at 61.5% (Figure 3). Some groupings were unexpected, such as a low‐bootstrap‐support grouping of TX2R, TX3R, TX4R and TX5R (Central Great Plains) populations with Alberta, Canada (Northern Plains), as well as OR1R (Figure 3). Populations KS13R, MT3R and CO6R clustered with this Pacific Northwest group (Figure 3) and had similarity in the structure analysis (Figure 4). The structure analysis showed that *K* = 3 was the best‐supported number of clusters or gene pools (Figure S1) and also supported the grouping of OR4R and MT2R with the Central Great Plains populations including CO1R, KS10R and KS11R (Figure 4). The PCA showed little population structure, with PC1 explaining 4.2% of the variation and PC2 explaining 3.9%, and no clear patterns of clustering by region were identified (Figure S2).

## 4 DISCUSSION

### 4.1 Evolutionary origin(s) of resistance

In our study, genomic‐based epidemiology using markers based on this first characterized *EPSPS* gene duplication variant from the Central Great Plains (referred to as genotype A) identified two additional *EPSPS* genotypes (genotypes B and C) in GR kochia populations from across western North America. Genotypes A (predominant in the Central Great Plains) and B (predominant in the Northern Plains) both showed increased copy numbers of the MGE previously identified, but genotype B does not have the MGE next to *EPSPS* at the same position as identified in genotype A, as shown by the lack of PCR amplification of type I and II repeats containing the MGE in genotype B individuals. *EPSPS* duplication in genotype B may have occurred through a similar genetic mechanism as genotype A, involving insertion of an active (but unique) MGE next to *EPSPS* causing double‐strand break and subsequent tandem duplication. Alternatively, genotype B may be derived from genotype A with loss of the type I and type II primer sites due to mutation accumulation over time. Resequencing and assembly of the *EPSPS* locus in genotype B will be needed to determine the precise duplication mechanism that occurred and to determine whether the MGE, which had increased copy number in genotype B, is associated with the *EPSPS* duplication and whether genotype B represents an independent origin of glyphosate resistance or not. In contrast, we consider genotype C (predominant in the Pacific Northwest) to be a convincing case of independent evolutionary origin of *EPSPS* gene duplication because it had increased *EPSPS* copy number and no increase in MGE copy number. An *EPSPS* duplication may also have occurred through a double‐strand break, but initiated by a different MGE than the one identified in the Central Great Plains populations, or perhaps a different mechanism of gene duplication occurred. These genotypes, based on either the presence or the absence of type I and type II elements and copy number of the MGE, provide evidence that glyphosate resistance has evolved at least twice independently in geographically distinct locations, with two independent origins supported by the data and the possibility of a third independent origin to be investigated. Determining whether genotype C, the event not associated with increased MGE copy number, occurred independently from genotype A via a different molecular genetic mechanism will require additional sequencing to assemble this specific *EPSPS* duplication variant.

The first report of GR kochia was from Kansas in 2007 (Waite et al., 2013) and reports have since confirmed glyphosate resistance in multiple US states and Canadian provinces (Kumar et al., 2019). The widespread regional evolution of GR kochia has negatively impacted the sustainability of reduced‐tillage weed management and moisture and soil conservation during fallow periods in the Central Great Plains of North America (Kumar et al., 2019). The mechanism of glyphosate resistance has been thoroughly investigated in kochia, in terms of physiology and fitness penalty as well as the genetic mechanisms that cause resistance (Beckie et al., 2018; Kumar & Jha, 2015; Martin et al., 2017; Osipitan & Dille, 2017; Wiersma et al., 2015). The first populations characterized for the molecular genetics of the resistance allele were from the Central Great Plains region (Kansas and Colorado) and had tandem *EPSPS* gene duplications that occurred at a single locus (Jugulam et al., 2014; Patterson et al., 2019). An increased copy number of the *EPSPS* gene has been identified as the resistance mechanism in all studied kochia populations to date from across seven US states (Montana, Wyoming, Oregon, Idaho, Nebraska, Kansas and Colorado) and in Canada (Gaines et al., 2016; Godar et al., 2015; Kumar et al., 2015, 2018; Martin et al., 2017; Wiersma et al., 2015).

### 4.2 Genomic mechanisms underlying resistant copy number variants

Mobile genetic elements can generate novel structural genomic variation through removal and/or insertion at new loci (Bennetzen, 2005; Stapley et al., 2015), which can result in adaptive genetic changes in plant (Li et al., 2018) and insect (Schmidt et al., 2010) populations. We assessed the structure of MGE presence or absence next to *EPSPS* in GR and GS kochia individuals. Neither type I nor type II markers were present in any of the evaluated GS kochia samples. PCR amplification of both type I and type II primers requires the presence of the MGE next to the *EPSPS* gene. The lack of PCR amplification confirms that the MGE is not present next to the *EPSPS* gene in any of the susceptible individuals sampled. The four to six copies of the MGE present in most GS individuals are located elsewhere in the genome and the MGE is not present next to the *EPSPS* gene in wild‐type kochia (Patterson et al., 2019). The MGE found next to *EPSPS* therefore must have a different ancestral location in the kochia genome. This further supports the hypothesis that MGE insertion occurred prior to the *EPSPS* duplication event and perhaps was even a trigger to initiate tandem duplication, rather than the MGE having been present next to *EPSPS* ancestrally and coduplicated with *EPSPS* (Patterson et al., 2019). The increase in MGE copy number in a few GS individuals (Figure 2) suggests that copy number of this MGE varies across populations and it may represent an active element, or that the *EPSPS* duplication variant can segregate away from other MGE duplication sites in the genome.

### 4.3 Gene flow of resistant genotypes

We utilized *EPSPS* duplication genotype to interpret patterns of gene flow among populations. Genotype C (increased *EPSPS* gene copy number without increased MGE copy number) was found in populations from Oregon, Idaho and Wyoming. Due to the lack of PCR amplification of the type I and II markers, interpreting whether these populations have independent origins or represent gene flow is challenging. From the neighbour‐joining tree, population WY1R (Northern Plains) was closest to OR5R (Pacific Northwest) with support of 41% (Figure 3), but this pair was distant from the other Pacific Northwest populations and not clustered with Pacific Northwest populations in the structure plot (Figure 4). The shared genotype C between geographically isolated northern Wyoming and the Pacific Northwest could indicate two separate origins of genotype C, or it could indicate lack of resolution in the population genetics data to resolve gene flow from independent origins of resistance. Characterization of the sequence variation at the duplicated resistance locus is needed to determine whether populations from the Northern Plains (WY1R) and the Pacific Northwest have a shared or separate origin of duplicated *EPSPS* genes.

The presence of more than one *EPSPS* genotype within populations provides evidence for gene flow among populations. Some Northern Plains populations (Wyoming and southern Montana) contained individuals with genotype B as well as individuals with genotype C (Table 3), indicating gene flow via migration between populations and/or dynamic MGE changes in copy number over time. This was observed in some Colorado populations, with some genotype B individuals present in populations that were mostly genotype A (e.g., CO3R from Cope, Colorado, showing high *EPSPS*, no type I or II, and high MGE, like samples from northern Montana and Alberta); alternatively, the presence of populations with both genotypes A and B could support the hypothesis that genotype B derived from genotype A through mutations in the PCR primer sites for the type I and II markers. Some genotype C individuals were present in populations mostly containing genotype A (Eaton, Colorado) (Figure 1, Table 3; Table S1). These two populations showing admixture in Colorado indicates gene flow has occurred in this area.

We predicted that population genetics analysis would show clear geographical structure if independent glyphosate resistance evolutionary events occurred and were followed by rapid dispersal and introgression within a region. Aside from a large grouping of Central Great Plains populations and a second grouping of Pacific Northwest populations, we were not able to identify clear geographical structure for the three regions corresponding to the three *EPSPS* genotypes. This lack of population and geographical structure is also supported by a PCA (Figure S2). This aligns with previous population genetics studies in kochia that have found high genetic diversity within individuals and little population structure (Friesen et al., 2009; Kumar et al., 2019; Mengistu & Messersmith, 2002). A recent study using high‐coverage single‐nucleotide variant data found almost no population structure (Martin et al., 2020), concluding that high gene flow rates occur across GR kochia populations. Although some strong signals of relatedness were detected between populations from geographically isolated locations, such as OR4R and KS10R (Figures 3 and 4), we consider it to be unlikely that the same duplication mechanism is evolving independently multiple times within a region on different genetic backgrounds. Instead, it is more likely the SSR data have insufficient resolution to identify population genetic relationships, and extensive long‐distance gene flow via seeds may make regional differences harder to detect. Whereas the structure analysis supports three groups, populations were not consistently assigned to three groups corresponding geographically to the regions containing the three *EPSPS* genotypes and most populations contained some presence of all three structure groups. This further supports the weak population structure found in the PCA. Kochia has protogynous flowers in which the stigmas emerge first and are receptive to pollen from other flowers before pollen production within the same flower occurs, reducing the self‐pollination rate (Blackwell & Powell, 1981; Guttieri et al., 1995). Additionally, kochia is a well‐known tumbleweed species, with some plants dispersing seeds for dozens or even hundreds of miles (Kumar et al., 2019). This dispersal mechanism greatly increases the spread of herbicide‐resistance alleles and makes containment extremely difficult (Beckie et al., 2016; Kumar et al., 2019; Stallings et al., 1995). Further research will be needed to quantify long‐distance gene flow in kochia, and the relative contributions of natural tumbling dispersal and human‐mediated seed migration. Palmer amaranth (*Amaranthus palmeri*), an obligate outcrossing weed species subjected to widespread glyphosate selection pressure, also maintains high levels of genetic diversity and little population structure.

### 4.4 Evolutionary origin(s) and gene flow of resistance in other species

Single origins of herbicide resistance followed by substantial geographical distribution by pollen‐ and seed‐mediated gene flow is known to have a major contribution to resistance frequency in several weed species (Beckie et al., 2019). The high sequence similarity of the extrachromosomal DNA containing the *EPSPS* gene in Palmer amaranth (Koo et al., 2018; Molin, Yaguchi, et al., 2020) across widespread populations supports the hypothesis of a single origin followed by dispersal (Molin, Patterson, et al., 2020; Molin et al., 2017, 2018). GR populations of flaxleaf fleabane (*Erigeron bonariensis*) from across multiple Australian states were highly related, supporting a single origin of resistance followed by a high frequency of seed movement (Minati et al., 2020). In contrast to these single‐origin examples, multiple independent origins of glyphosate resistance with little population structure were found in GR populations of common morning glory (*Ipomoea purpurea*) (Kuester et al., 2015). A genomics‐based approach in the same species found evidence for parallel genetic responses in genomic regions encoding potential herbicide detoxification enzymes, while other genomic regions showed divergent patterns of selection (Van Etten et al., 2020). Convergent evolution of *EPSPS* gene duplication with unique structural variation was found in waterhemp (*Amaranthus tuberculatus*) from the US Midwest and Ontario, Canada, suggesting at least two independent evolutionary origins of resistance alleles followed by gene flow and introgression into two populations (Kreiner et al., 2019). Multiple independent origins of glyphosate resistance were also detected in horseweed (*Erigeron canadensis*) in California, with localized movement of resistant individuals accounting for spread on regional levels correlating with groundwater regulations that encouraged more glyphosate use and less use of other herbicides (Okada et al., 2013).

In summary, we have used genomic‐based epidemiology to track the mutations underlying one specific origin of glyphosate resistance in kochia and showed that at least two independent origins of glyphosate resistance have evolved in kochia, followed by substantial regional gene flow to spread the resistance alleles to new genetic backgrounds. Due to the tumbling dispersal of kochia, intercepting seed movement across the landscape has high potential to mitigate the negative impact of herbicide resistance spreading from an initial origin. With the kochia reference genome now available (Patterson et al., 2019), the population genomics approach used by Kreiner et al. (2019) can be used in kochia to study population divergence and origins of resistance (Martin et al., 2019). From the results of this study, we will continue to investigate the hypothesis that genotype C represents an independent origin and the hypothesis that genotype B is either an independent origin or derived from genotype A with loss of PCR primer sites for the type I and type II markers. We will test these hypotheses by sequencing and assembling the duplicated region from individuals with genotypes B and C to provide insights and new markers to further investigate the evolutionary dynamics of the *EPSPS* tandem duplication in kochia across western North America.

## AUTHOR CONTRIBUTIONS

K.R., C.S., A.D., P.N., E.P., P.W. and T.G. designed research; K.R., C.D., A.D., A.K., E.W., D.P., P.T., J.F., D.W., P.J., A.K., P.S. and E.P. performed research; K.R., C.S., A.D., A.K., E.P., A.K. and T.G. analysed research; K.R., C.S., A.D., A.K., E.P., P.W. and T.G. wrote the paper; all authors contributed to editing and approved the final version of the paper.

## CONFLICT OF INTEREST

The authors declare no conflict of interest.

## Supporting information

Supplementary Material

## Data Availability

SSR genotypic data and R scripts used for population genetics analysis and qPCR data for *EPSPS*, Type I, Type II and MGE copy numbers are available at the digital repository Mountain Scholar, https://doi.org/10.25675/10217/216180.

## References

[mec16215-bib-0001] Amos, B. (2005). FlexiBin, a program to automate the binning of microsatellite alleles. Retrieved from https://www.zoo.cam.ac.uk/research‐groups/molecular‐ecology/FlexiBin.pdf

[mec16215-bib-0002] Amos, W. , Hoffman, J. , Frodsham, A. , Zhang, L. , Best, S. , & Hill, A. (2007). Automated binning of microsatellite alleles: Problems and solutions. Molecular Ecology Notes, 7, 10–14. 10.1111/j.1471-8286.2006.01560.x

[mec16215-bib-0003] Barrett, R. D. , & Schluter, D. (2008). Adaptation from standing genetic variation. Trends in Ecology & Evolution, 23, 38–44. 10.1016/j.tree.2007.09.008 18006185

[mec16215-bib-0004] Baucom, R. S. (2019). Evolutionary and ecological insights from herbicide‐resistant weeds: What have we learned about plant adaptation, and what is left to uncover? New Phytologist, 223, 68–82. 30710343 10.1111/nph.15723

[mec16215-bib-0005] Beckie, H. J. , Blackshaw, R. E. , Hall, L. M. , & Johnson, E. N. (2016). Pollen‐and seed‐mediated gene flow in kochia (*Kochia scoparia*). Weed Science, 64, 624–633.

[mec16215-bib-0006] Beckie, H. J. , Blackshaw, R. E. , Leeson, J. , Stahlman, P. W. , Gaines, T. , & Johnson, E. A. (2018). Seed bank persistence, germination and early growth of glyphosate‐resistant *Kochia scoparia* . Weed Research, 58, 177–187.

[mec16215-bib-0007] Beckie, H. J. , Busi, R. , Bagavathiannan, M. V. , & Martin, S. L. (2019). Herbicide resistance gene flow in weeds: Under‐estimated and under‐appreciated. Agriculture, Ecosystems & Environment, 283, 106566. 10.1016/j.agee.2019.06.005

[mec16215-bib-0008] Beckie, H. J. , Busi, R. , Lopez‐Ruiz, F. J. , & Umina, P. A. (2021). Herbicide resistance management strategies: How do they compare with those for insecticides, fungicides and antibiotics? Pest Management Science, 77, 3049–3056. 33821561 10.1002/ps.6395

[mec16215-bib-0009] Bennetzen, J. L. (2005). Transposable elements, gene creation and genome rearrangement in flowering plants. Current Opinion in Genetics & Development, 15, 621–627. 10.1016/j.gde.2005.09.010 16219458

[mec16215-bib-0010] Benvenuti, S. (2007). Weed seed movement and dispersal strategies in the agricultural environment. Weed Biology and Management, 7, 141–157. 10.1111/j.1445-6664.2007.00249.x

[mec16215-bib-0011] Blackwell, W. H. , & Powell, M. J. (1981). A preliminary note on pollination in the Chenopodiaceae. Annals of the Missouri Botanical Garden, 68, 524–526. 10.2307/2398886

[mec16215-bib-0012] Délye, C. , Deulvot, C. , & Chauvel, B. (2013). DNA analysis of herbarium specimens of the grass weed *Alopecurus myosuroides* reveals herbicide resistance pre‐dated herbicides. PLoS One, 8, e75117. 10.1371/journal.pone.0075117 24146749 PMC3797703

[mec16215-bib-0013] Délye, C. , Michel, S. , Bérard, A. , Chauvel, B. , Brunel, D. , Guillemin, J.‐P. , & Le Corre, V. (2010). Geographical variation in resistance to acetyl‐coenzyme A carboxylase‐inhibiting herbicides across the range of the arable weed *Alopecurus myosuroides* (black‐grass). New Phytologist, 186, 1005–1017. 20345631 10.1111/j.1469-8137.2010.03233.x

[mec16215-bib-0014] Dixon, A. , Comont, D. , Slavov, G. T. , & Neve, P. (2021). Population genomics of selectively neutral genetic structure and herbicide resistance in UK populations of *Alopecurus myosuroides* . Pest Management Science, 77, 1520–1529. 33155426 10.1002/ps.6174

[mec16215-bib-0015] Doyle, J. (1991). DNA protocols for plants–CTAB total DNA isolation. In G. M. Hewitt & A. Johnston (Eds.), Molecular techniques in taxonomy (pp. 283–293). Springer.

[mec16215-bib-0016] Dray, S. , & Dufour, A.‐B. (2007). The ade4 package: Implementing the duality diagram for ecologists. Journal of Statistical Software, 22, 1–20.

[mec16215-bib-0017] Earl, D. A. , & vonHoldt, B. M. (2012). STRUCTURE HARVESTER: A website and program for visualizing STRUCTURE output and implementing the Evanno method. Conservation Genetics Resources, 4, 359–361. 10.1007/s12686-011-9548-7

[mec16215-bib-0018] Evanno, G. , Regnaut, S. , & Goudet, J. (2005). Detecting the number of clusters of individuals using the software STRUCTURE: A simulation study. Molecular Ecology, 14, 2611–2620. 10.1111/j.1365-294X.2005.02553.x 15969739

[mec16215-bib-0019] Friesen, L. F. , Beckie, H. J. , Warwick, S. I. , & Van Acker, R. C. (2009). The biology of Canadian weeds. 138. *Kochia scoparia* (L.) Schrad. Canadian Journal of Plant Science, 89, 141–167.

[mec16215-bib-0020] Gaines, T. A. , Barker, A. L. , Patterson, E. L. , Westra, P. , Westra, E. P. , Wilson, R. G. , Jha, P. , Kumar, V. , & Kniss, A. R. (2016). *EPSPS* gene copy number and whole‐plant glyphosate resistance level in *Kochia scoparia* . PLoS One, 11, e0168295. 10.1371/journal.pone.0168295 27992501 PMC5161467

[mec16215-bib-0021] Gaines, T. A. , Duke, S. O. , Morran, S. , Rigon, C. A. G. , Tranel, P. J. , Küpper, A. , & Dayan, F. E. (2020). Mechanisms of evolved herbicide resistance. Journal of Biological Chemistry, 295, 10307–10330. 10.1074/jbc.REV120.013572 32430396 PMC7383398

[mec16215-bib-0022] Godar, A. S. , Stahlman, P. W. , Jugulam, M. , & Dille, J. A. (2015). Glyphosate‐resistant kochia (*Kochia scoparia*) in Kansas: EPSPS gene copy number in relation to resistance levels. Weed Science, 63, 587–595.

[mec16215-bib-0023] Guttieri, M. J. , Eberlein, C. V. , & Thill, D. C. (1995). Diverse mutations in the acetolactate synthase gene confer chlorsulfuron resistance in kochia (*Kochia scoparia*) biotypes. Weed Science, 43, 175–178.

[mec16215-bib-0024] Hawkins, N. J. , Bass, C. , Dixon, A. , & Neve, P. (2019). The evolutionary origins of pesticide resistance. Biological Reviews, 94, 135–155. 10.1111/brv.12440 PMC6378405 29971903

[mec16215-bib-0025] Heap, I. (2020). The international survey of herbicide resistant weeds. Retrieved from www.weedscience.com

[mec16215-bib-0026] Hermisson, J. , & Pennings, P. S. (2005). Soft sweeps: Molecular population genetics of adaptation from standing genetic variation. Genetics, 169, 2335–2352. 10.1534/genetics.104.036947 15716498 PMC1449620

[mec16215-bib-0027] Jakobsson, M. , & Rosenberg, N. A. (2007). CLUMPP: A cluster matching and permutation program for dealing with label switching and multimodality in analysis of population structure. Bioinformatics, 23, 1801–1806. 10.1093/bioinformatics/btm233 17485429

[mec16215-bib-0028] Jombart, T. , & Ahmed, I. (2011). adegenet 1.3‐1: New tools for the analysis of genome‐wide SNP data. Bioinformatics, 27, 3070–3071. 10.1093/bioinformatics/btr521 21926124 PMC3198581

[mec16215-bib-0029] Jugulam, M. , Niehues, K. , Godar, A. S. , Koo, D.‐H. , Danilova, T. , Friebe, B. , & Gill, B. S. (2014). Tandem amplification of a chromosomal segment harboring EPSPS locus confers glyphosate resistance in *Kochia scoparia* . Plant Physiology, 166, 1200–1207. 25037215 10.1104/pp.114.242826 PMC4226373

[mec16215-bib-0030] Kamvar, Z. N. , Brooks, J. C. , & Grünwald, N. J. (2015). Novel R tools for analysis of genome‐wide population genetic data with emphasis on clonality. Frontiers in Genetics, 6, 208. 10.3389/fgene.2015.00208 26113860 PMC4462096

[mec16215-bib-0031] Kamvar, Z. N. , Tabima, J. F. , & Grünwald, N. J. (2014). Poppr: An R package for genetic analysis of populations with clonal, partially clonal, and/or sexual reproduction. PeerJ, 2, e281. 24688859 10.7717/peerj.281 PMC3961149

[mec16215-bib-0032] Keenan, K. , McGinnity, P. , Cross, T. F. , Crozier, W. W. , & Prodöhl, P. A. (2013). diveRsity: An R package for the estimation and exploration of population genetics parameters and their associated errors. Methods in Ecology and Evolution, 4, 782–788.

[mec16215-bib-0033] Koo, D.‐H. , Molin, W. T. , Saski, C. A. , Jiang, J. , Putta, K. , Jugulam, M. , & Gill, B. S. (2018). Extrachromosomal circular DNA‐based amplification and transmission of herbicide resistance in crop weed *Amaranthus palmeri* . Proceedings of the National Academy of Sciences of the United States of America, 115, 3332–3337. 29531028 10.1073/pnas.1719354115 PMC5879691

[mec16215-bib-0034] Kreiner, J. M. , Giacomini, D. A. , Bemm, F. , Waithaka, B. , Regalado, J. , Lanz, C. , & Wright, S. I. (2019). Multiple modes of convergent adaptation in the spread of glyphosate‐resistant *Amaranthus tuberculatus* . Proceedings of the National Academy of Sciences of the United States of America, 116, 21076–21084. 31570613 10.1073/pnas.1900870116 PMC6800383

[mec16215-bib-0035] Kreiner, J. M. , Stinchcombe, J. R. , & Wright, S. I. (2018). Population genomics of herbicide resistance: Adaptation via evolutionary rescue. Annual Review of Plant Biology, 69, 611–635. 10.1146/annurev-arplant-042817-040038 29140727

[mec16215-bib-0036] Kuester, A. , Chang, S.‐M. , & Baucom, R. S. (2015). The geographic mosaic of herbicide resistance evolution in the common morning glory, *Ipomoea purpurea*: Evidence for resistance hotspots and low genetic differentiation across the landscape. Evolutionary Applications, 8, 821–833. 26366199 10.1111/eva.12290 PMC4561571

[mec16215-bib-0037] Kumar, V. , Felix, J. , Morishita, D. , & Jha, P. (2018). Confirmation of glyphosate‐resistant kochia (*Kochia scoparia*) from sugar beet fields in Idaho and Oregon. Weed Technology, 32, 27–33.

[mec16215-bib-0038] Kumar, V. , & Jha, P. (2015). Growth and reproduction of glyphosate‐resistant and susceptible populations of *Kochia scoparia* . PLoS One, 10, e0142675. 10.1371/journal.pone.0142675 26580558 PMC4651312

[mec16215-bib-0039] Kumar, V. , Jha, P. , Giacomini, D. , Westra, E. P. , & Westra, P. (2015). Molecular basis of evolved resistance to glyphosate and acetolactate synthase‐inhibitor herbicides in kochia (*Kochia scoparia*) accessions from Montana. Weed Science, 63, 758–769.

[mec16215-bib-0040] Kumar, V. , Jha, P. , Jugulam, M. , Yadav, R. , & Stahlman, P. W. (2019). Herbicide‐resistant kochia (*Bassia scoparia*) in North America: A review. Weed Science, 67, 4–15.

[mec16215-bib-0041] Kumar, V. , Jha, P. , & Reichard, N. (2014). Occurrence and characterization of kochia (*Kochia scoparia*) accessions with resistance to glyphosate in Montana. Weed Technology, 28, 122–130.

[mec16215-bib-0042] Lee, R. M. , Thimmapuram, J. , Thinglum, K. A. , Gong, G. , Hernandez, A. G. , Wright, C. L. , & Tranel, P. J. (2009). Sampling the waterhemp (*Amaranthus tuberculatus*) genome using pyrosequencing technology. Weed Science, 57, 463–469.

[mec16215-bib-0043] Letunic, I. , & Bork, P. (2019). Interactive Tree Of Life (iTOL) v4: Recent updates and new developments. Nucleic Acids Research, 47, W256–W259. 10.1093/nar/gkz239 30931475 PMC6602468

[mec16215-bib-0044] Li, Z.‐W. , Hou, X.‐H. , Chen, J.‐F. , Xu, Y.‐C. , Wu, Q. , González, J. , & Guo, Y.‐L. (2018). Transposable elements contribute to the adaptation of *Arabidopsis thaliana* . Genome Biology and Evolution, 10, 2140–2150. 10.1093/gbe/evy171 30102348 PMC6117151

[mec16215-bib-0045] Martin, S. L. , Benedict, L. , Sauder, C. A. , Wei, W. , da Costa, L. O. , Hall, L. M. , & Beckie, H. J. (2017). Glyphosate resistance reduces kochia fitness: Comparison of segregating resistant and susceptible F2 populations. Plant Science, 261, 69–79. 10.1016/j.plantsci.2017.04.010 28554695

[mec16215-bib-0046] Martin, S. L. , Benedict, L. , Wei, W. , Sauder, C. A. , Beckie, H. J. , & Hall, L. M. (2020). High gene flow maintains genetic diversity following selection for high *EPSPS* copy number in the weed kochia (Amaranthaceae). Scientific Reports, 10, 1–11. 10.1038/s41598-020-75345-6 33139774 PMC7608611

[mec16215-bib-0047] Martin, S. L. , Parent, J.‐S. , Laforest, M. , Page, E. , Kreiner, J. M. , & James, T. (2019). Population genomic approaches for weed science. Plants, 8, 354. 10.3390/plants8090354 PMC6783936 31546893

[mec16215-bib-0048] Menchari, Y. , Camilleri, C. , Michel, S. , Brunel, D. , Dessaint, F. , Le Corre, V. , & Délye, C. (2006). Weed response to herbicides: Regional‐scale distribution of herbicide resistance alleles in the grass weed *Alopecurus myosuroides* . New Phytologist, 171, 861–874. 16918556 10.1111/j.1469-8137.2006.01788.x

[mec16215-bib-0049] Mengistu, L. W. , & Messersmith, C. G. (2002). Genetic diversity of kochia. Weed Science, 50, 498–503.

[mec16215-bib-0050] Messer, P. W. , & Petrov, D. A. (2013). Population genomics of rapid adaptation by soft selective sweeps. Trends in Ecology & Evolution, 28, 659–669. 10.1016/j.tree.2013.08.003 24075201 PMC3834262

[mec16215-bib-0051] Minati, M. H. , Preston, C. , & Malone, J. (2020). Genetic diversity and spread of glyphosate‐resistant flaxleaf fleabane. Bulletin of the National Research Centre, 44, 24. 10.1186/s42269-020-0277-5

[mec16215-bib-0052] Molin, W. T. , Patterson, E. L. , & Saski, C. A. (2020). Homogeneity among glyphosate‐resistant *Amaranthus palmeri* in geographically distant locations. PLoS One, 15, e0233813. 32903277 10.1371/journal.pone.0233813 PMC7480871

[mec16215-bib-0053] Molin, W. T. , Wright, A. A. , Lawton‐Rauh, A. , & Saski, C. A. (2017). The unique genomic landscape surrounding the *EPSPS* gene in glyphosate resistant *Amaranthus palmeri*: A repetitive path to resistance. Bmc Genomics, 18, 91. 10.1186/s12864-016-3336-4 28095770 PMC5240378

[mec16215-bib-0054] Molin, W. T. , Wright, A. A. , VanGessel, M. J. , McCloskey, W. B. , Jugulam, M. , & Hoagland, R. E. (2018). Survey of the genomic landscape surrounding the 5‐enolpyruvylshikimate‐3‐phosphate synthase (*EPSPS*) gene in glyphosate‐resistant *Amaranthus palmeri* from geographically distant populations in the United States. Pest Management Science, 74, 1109–1117. 28686355 10.1002/ps.4659

[mec16215-bib-0055] Molin, W. T. , Yaguchi, A. , Blenner, M. A. , & Saski, C. A. (2020). The eccDNA Replicon: A heritable, extra‐nuclear vehicle that enables gene amplification and glyphosate resistance in *Amaranthus palmeri* . The Plant Cell, 32, 2132–2140. 32327538 10.1105/tpc.20.00099 PMC7346551

[mec16215-bib-0056] Neve, P. , Busi, R. , Renton, M. , & Vila‐Aiub, M. M. (2014). Expanding the eco‐evolutionary context of herbicide resistance research. Pest Management Science, 70, 1385–1393. 10.1002/ps.3757 24723489

[mec16215-bib-0057] Okada, M. , Hanson, B. D. , Hembree, K. J. , Peng, Y. , Shrestha, A. , Stewart, C. N. Jr , & Jasieniuk, M. (2013). Evolution and spread of glyphosate resistance in *Conyza canadensis* in California. Evolutionary Applications, 6, 761–777. 29387164 10.1111/eva.12061 PMC5779124

[mec16215-bib-0058] Osipitan, O. A. , & Dille, J. A. (2017). Fitness outcomes related to glyphosate resistance in kochia (*Kochia scoparia*): What life history stage to examine? Frontiers in Plant Science, 8, 1090. 28713397 10.3389/fpls.2017.01090 PMC5491607

[mec16215-bib-0059] Patterson, E. L. , Saski, C. A. , Sloan, D. B. , Tranel, P. J. , Westra, P. , & Gaines, T. A. (2019). The draft genome of *Kochia scoparia* and the mechanism of glyphosate resistance via transposon‐mediated *EPSPS* tandem gene duplication. Genome Biology and Evolution, 11, 2927–2940. 10.1093/gbe/evz198 31518388 PMC6808082

[mec16215-bib-0060] Pritchard, J. K. , Stephens, M. , & Donnelly, P. (2000). Inference of population structure using multilocus genotype data. Genetics, 155, 945–959. 10.1093/genetics/155.2.945 10835412 PMC1461096

[mec16215-bib-0061] R Core Team . (2019). R: A language and environment for statistical computing. R Foundation for Statistical Computing. Retrieved from http://www.R‐project/org/

[mec16215-bib-0062] Rosenberg, N. A. (2004). DISTRUCT: A program for the graphical display of population structure. Molecular Ecology Notes, 4, 137–138. 10.1046/j.1471-8286.2003.00566.x

[mec16215-bib-0063] Rousset, F. (2008). genepop’007: A complete re‐implementation of the genepop software for Windows and Linux. Molecular Ecology Resources, 8, 103–106. 10.1111/j.1471-8286.2007.01931.x 21585727

[mec16215-bib-0064] Schmidt, J. M. , Good, R. T. , Appleton, B. , Sherrard, J. , Raymant, G. C. , Bogwitz, M. R. , Martin, J. , Daborn, P. J. , Goddard, M. E. , Batterham, P. , & Robin, C. (2010). Copy number variation and transposable elements feature in recent, ongoing adaptation at the Cyp6g1 locus. PLoS Genetics, 6, e1000998. 10.1371/journal.pgen.1000998 20585622 PMC2891717

[mec16215-bib-0065] Schmittgen, T. D. , & Livak, K. J. (2008). Analyzing real‐time PCR data by the comparative C_T_ method. Nature Protocols, 3, 1101–1108. 10.1038/nprot.2008.73 18546601

[mec16215-bib-0066] Stallings, G. P. , Thill, D. C. , Mallory‐Smith, C. A. , & Shafii, B. (1995). Pollen‐mediated gene flow of sulfonylurea‐resistant kochia (*Kochia scoparia*). Weed Science, 43, 95–102.

[mec16215-bib-0067] Stapley, J. , Santure, A. W. , & Dennis, S. R. (2015). Transposable elements as agents of rapid adaptation may explain the genetic paradox of invasive species. Molecular Ecology, 24, 2241–2252. 10.1111/mec.13089 25611725

[mec16215-bib-0068] Van Etten, M. , Lee, K. M. , Chang, S.‐M. , & Baucom, R. S. (2020). Parallel and nonparallel genomic responses contribute to herbicide resistance in *Ipomoea purpurea*, a common agricultural weed. PLoS Genetics, 16, e1008593. 10.1371/journal.pgen.1008593 32012153 PMC7018220

[mec16215-bib-0069] Vila‐Aiub, M. M. , Neve, P. , & Powles, S. B. (2009). Fitness costs associated with evolved herbicide resistance alleles in plants. New Phytologist, 184, 751–767. 10.1111/j.1469-8137.2009.03055.x 19825013

[mec16215-bib-0070] Waite, J. , Thompson, C. R. , Peterson, D. E. , Currie, R. S. , Olson, B. L. S. , Stahlman, P. W. , & Al‐Khatib, K. (2013). Differential kochia (*Kochia scoparia*) populations response to glyphosate. Weed Science, 61, 193–200.

[mec16215-bib-0071] Waples, R. S. (2015). Testing for Hardy‐Weinberg proportions: Have we lost the plot? Journal of Heredity, 106, 1–19. 25425676 10.1093/jhered/esu062

[mec16215-bib-0072] Westra, E. P. , Nissen, S. J. , Getts, T. J. , Westra, P. , & Gaines, T. A. (2019). Survey reveals frequency of multiple resistance to glyphosate and dicamba in kochia (*Bassia scoparia*). Weed Technology, 33, 664–672.

[mec16215-bib-0073] Wiersma, A. T. , Gaines, T. A. , Preston, C. , Hamilton, J. P. , Giacomini, D. , Robin Buell, C. , Leach, J. E. , & Westra, P. (2015). Gene amplification of 5‐enol‐pyruvylshikimate‐3‐phosphate synthase in glyphosate‐resistant *Kochia scoparia* . Planta, 241, 463–474. 10.1007/s00425-014-2197-9 25366557

