# Supplementary material for: Genomic‐based epidemiology reveals independent origins and gene flow of glyphosate resistance in Bassia scoparia populations across North America
Source: Mol Ecol. 2021 Oct 21;30(21):5343–59. doi: 10.1111/mec.16215 (PMC9297870; doi:10.1111/mec.16215)
Supplement: Supplementary file 1 — Supplementary Material [file MEC-30-5343-s001.docx]

**Supplemental Information for:**

**Genomic-based epidemiology reveals gene flow and independent origins of glyphosate resistance in *Bassia scoparia* populations across North America**

Karl Ravet, Crystal D. Sparks, Andrea L. Dixon, Anita Küpper, Eric P. Westra, Dean J. Pettinga, Patrick J. Tranel, Joel Felix, Don W. Morishita, Prashant Jha, Andrew Kniss, Phillip W. Stahlman, Paul Neve, Eric L. Patterson, Philip Westra, and Todd A. Gaines

**Table of Contents:**

| **Table S1** | Page 2-8 |
| --- | --- |
| **Table S2** | Page 9 |
| **Table S3** | Page 10-11 |
| **Figure S1** | Page 12 |
| **Figure S2** | Page 13 |

**Table S1.** Genomic copy number of *EPSPS*, Type I and II repeats, and Mobile Genetic Element (MGE) in kochia (*Bassia scoparia*) populations from the western US and Canada shows three haplotypes, suggesting at least three independent origins of evolution of *EPSPS* gene duplication. Some populations contained individuals with no increase in *EPSPS* copy number and no change in MGE, shown at end of table. Genomic DNA from 113 individuals representing 27 of the populations used for the SSR markers, along with 36 individuals representing 11 populations used in Gaines et al. (2016) (designated SBK) and 58 individuals representing 15 populations collected in Montana (Population ID n/a). Three categories of *EPSPS*-region haplotypes were defined; 1) extra *EPSPS*, Type I and II, extra MGE (Central Great Plains); 2) extra *EPSPS*, no Type I or II, extra MGE (Northern Plains); 3) extra *EPSPS*, no Type I or II, no extra MGE (Pacific Northwest, some Wyoming).

|  |  |  |  |  | Relative Copy Number | | | |
| --- | --- | --- | --- | --- | --- | --- | --- | --- |
| Haplotype | Used in SSR Study | Population | Location | Population/ County/City | EPSPS | Type I | Type II | MGE |
| Reference Lines | Yes | CO9S | Colorado | 7710 | 1.0 | 0.0 | 0.0 | 5.8 |
|  | Yes | CO9S | Colorado | 7710 | 1.1 | 0.0 | 0.0 | 5.8 |
|  | Yes | CO9S | Colorado | 7710 | 1.0 | 0.0 | 0.0 | 6.2 |
|  | Yes | CO1R | Control | M32/Akron | 12.0 | 12.1 | 4.0 | 22.6 |
|  | Yes | CO1R | Control | M32/Akron | 10.6 | 10.7 | 3.8 | 23.5 |
|  | Yes | CO1R | Control | M32/Akron | 10.3 | 12.9 | 4.1 | 23.9 |
|  | Yes | CO1R | Control | M32/Akron | 11.0 | 13.9 | 4.3 | 25.2 |
|  | Yes | CO1R | Control | M32/Akron | 7.3 | 9.6 | 3.0 | 17.3 |
| Haplotype 1 |  |  |  |  |  |  |  |  |
|  | Yes | OK1R | Oklahoma | Cimarron | 5.1 | 6.1 | 2.2 | 13.1 |
|  | Yes | OK1R | Oklahoma | Cimarron | 3.9 | 5.4 | 1.9 | 14.8 |
|  | Yes | OK1R | Oklahoma | Cimarron | 9.9 | 12.3 | 3.6 | 20.7 |
|  | Yes | OK1R | Oklahoma | Cimarron | 9.8 | 13.5 | 4.8 | 22.8 |
|  | Yes | OK1R | Oklahoma | Cimarron | 10.4 | 14.3 | 4.6 | 26.5 |
|  | Yes | CO4R | Colorado | Julesburg | 7.8 | 9.5 | 2.8 | 18.7 |
|  | Yes | CO4R | Colorado | Julesburg | 9.8 | 11.2 | 3.2 | 20.0 |
|  | Yes | CO4R | Colorado | Julesburg | 8.6 | 11.7 | 3.5 | 24.0 |
|  | Yes | KS3R | Kansas | Gray | 7.6 | 10.2 | 3.3 | 20.0 |
|  | Yes | KS3R | Kansas | Gray | 6.7 | 9.2 | 3.0 | 17.4 |
|  | Yes | KS3R | Kansas | Gray | 4.8 | 4.8 | 1.6 | 14.3 |
|  | Yes | KS3R | Kansas | Gray | 5.5 | 5.4 | 0.9 | 15.2 |
|  | Yes | KS3R | Kansas | Gray | 9.9 | 10.4 | 3.0 | 17.2 |
|  | Yes | KS3R | Kansas | Gray | 7.8 | 9.2 | 2.8 | 15.6 |
|  | Yes | KS4R | Kansas | Greeley | 2.8 | 5.6 | 1.8 | 12.2 |
|  | Yes | KS4R | Kansas | Greeley | 4.0 | 6.3 | 1.9 | 14.9 |
|  | Yes | KS4R | Kansas | Greeley | 4.2 | 6.2 | 2.2 | 15.6 |
|  | Yes | KS4R | Kansas | Greeley | 23.1 | 23.1 | 12.0 | 11.6 |
|  | Yes | TX2R | Texas | Hartley | 7.0 | 8.6 | 3.1 | 18.1 |
|  | Yes | TX2R | Texas | Hartley | 6.5 | 9.2 | 3.7 | 20.7 |
|  | Yes | TX2R | Texas | Hartley | 9.3 | 10.1 | 4.6 | 25.9 |
|  | Yes | TX3R | Texas | Hartley | 4.7 | 4.7 | 1.8 | 12.2 |
|  | Yes | TX3R | Texas | Hartley | 4.3 | 4.5 | 1.7 | 13.1 |
|  | Yes | TX3R | Texas | Hartley | 7.0 | 4.5 | 1.6 | 14.2 |
|  | Yes | TX4R | Texas | Hartley | 5.8 | 5.7 | 1.8 | 14.4 |
|  | Yes | TX4R | Texas | Hartley | 7.0 | 6.0 | 2.3 | 19.7 |
|  | Yes | TX4R | Texas | Hartley | 13.5 | 11.8 | 4.7 | 19.9 |
|  | Yes | TX4R | Texas | Hartley | 12.6 | 14.9 | 4.6 | 24.7 |
|  | Yes | TX5R | Texas | Hartley | 1.9 | 4.2 | 2.0 | 11.0 |
|  | Yes | TX5R | Texas | Hartley | 6.2 | 6.1 | 2.9 | 11.1 |
|  | Yes | TX5R | Texas | Hartley | 11.4 | 10.8 | 5.3 | 19.8 |
|  | Yes | TX5R | Texas | Hartley | 13.5 | 12.9 | 5.2 | 22.8 |
|  | Yes | TX5R | Texas | Hartley | 8.3 | 8.9 | 3.2 | 26.1 |
|  | Yes | CO5R | Colorado | Kit Carson | 4.0 | 5.8 | 1.6 | 13.2 |
|  | Yes | CO5R | Colorado | Kit Carson | 7.3 | 11.9 | 3.4 | 26.0 |
|  | Yes | CO5R | Colorado | Kit Carson | 9.5 | 12.7 | 4.1 | 34.4 |
|  | Yes | KS5S | Kansas | Meade | 5.8 | 7.2 | 2.1 | 15.3 |
|  | Yes | KS6S | Kansas | Ness | 4.4 | 5.3 | 2.0 | 12.1 |
|  | Yes | KS6S | Kansas | Ness | 2.8 | 6.3 | 1.7 | 14.7 |
|  | Yes | KS6S | Kansas | Ness | 2.5 | 6.2 | 1.9 | 16.1 |
|  | Yes | KS6S | Kansas | Ness | 2.4 | 6.2 | 2.2 | 17.6 |
|  | Yes | KS6S | Kansas | Ness | 10.2 | 11.4 | 4.1 | 21.3 |
|  | Yes | KS6S | Kansas | Ness | 6.4 | 12.9 | 4.2 | 25.6 |
|  | Yes | KS6S | Kansas | Ness | 5.5 | 13.7 | 4.1 | 27.3 |
|  | Yes | KS6S | Kansas | Ness | 4.2 | 12.0 | 3.7 | 28.5 |
|  | Yes | TX1R | Texas | Hartley | 7.9 | 5.6 | 2.1 | 17.4 |
|  | Yes | TX1R | Texas | Hartley | 9.9 | 12.4 | 3.8 | 24.4 |
|  | No | SBK-11 | Colorado | Mead | 6.8 | 10.5 | 3.1 | 20.0 |
|  | No | SBK-11 | Colorado | Mead | 8.7 | 12.3 | 3.1 | 21.0 |
|  | No | SBK-11 | Colorado | Mead | 9.5 | 12.1 | 3.0 | 21.8 |
|  | No | SBK-20 | Nebraska | Alliance | 6.2 | 6.7 | 2.2 | 12.4 |
|  | No | SBK-20 | Nebraska | Alliance | 10.2 | 12.6 | 4.2 | 26.4 |
|  | Yes | KS9R | Kansas | Scott | 9.7 | 9.7 | 4.0 | 3.4 |
|  | Yes | KS9R | Kansas | Scott | 2.0 | 2.0 | 3.6 | 2.9 |
|  | No | SBK-14 | Nebraska | Imperial | 4.4 | 5.5 | 1.6 | 10.0 |
|  | No | SBK-14 | Nebraska | Imperial | 4.1 | 5.6 | 1.7 | 14.8 |
|  | Yes | KS12R | Kansas | Thomas | 4.4 | 5.3 | 1.7 | 12.8 |
|  | Yes | KS12R | Kansas | Thomas | 3.8 | 5.6 | 1.8 | 11.6 |
|  | Yes | KS12R | Kansas | Thomas | 5.0 | 5.6 | 1.8 | 12.1 |
|  | Yes | KS12R | Kansas | Thomas | 4.9 | 5.7 | 1.9 | 12.1 |
|  | Yes | KS12R | Kansas | Thomas | 11.9 | 14.9 | 4.4 | 31.9 |
|  | Yes | KS12R | Kansas | Thomas | 8.7 | 11.5 | 3.9 | 22.2 |
| Haplotype 2 |  |  |  |  |  |  |  |  |
|  | No | n/a | Montana | Greg_P1R2(8/9) | 18.9 | 0.0 | 0.0 | 32.7 |
|  | No | n/a | Montana | Greg_P2R1(8/9) | 16.3 | 0.0 | 0.0 | 52.3 |
|  | No | n/a | Montana | Greg_P2R2(8/9) | 4.4 | 0.0 | 0.0 | 21.1 |
|  | No | n/a | Montana | Cropland_P1R1 | 31.3 | 0.0 | 0.0 | 95.0 |
|  | No | n/a | Montana | Cropland_P1R2 | 28.8 | 0.0 | 0.0 | 67.2 |
|  | No | n/a | Montana | Beaverton | 15.1 | 0.0 | 0.0 | 29.7 |
|  | No | n/a | Montana | Beaverton | 22.5 | 0.0 | 0.0 | 33.6 |
|  | No | n/a | Montana | C_8/1_R14 | 7.8 | 0.0 | 0.0 | 20.0 |
|  | No | n/a | Montana | C_8/2_R14 | 12.6 | 0.0 | 0.0 | 27.3 |
|  | No | n/a | Montana | C_8/3_R14 | 8.5 | 0.0 | 0.0 | 20.0 |
|  | No | n/a | Montana | C_8/4_R14 | 7.9 | 0.0 | 0.0 | 15.5 |
|  | No | n/a | Montana | C_8/5_R14 | 8.1 | 0.0 | 0.0 | 17.5 |
|  | No | n/a | Montana | C_8/6_R14 | 11.4 | 0.0 | 0.0 | 23.3 |
|  | No | n/a | Montana | C4_756_P1 | 6.5 | 0.0 | 0.0 | 11.6 |
|  | No | n/a | Montana | Cut Bank | 8.4 | 0.0 | 0.0 | 22.8 |
|  | No | n/a | Montana | Haven1_K1 | 13.1 | 0.0 | 0.0 | 40.2 |
|  | No | n/a | Montana | Haven1_K2 | 13.6 | 0.0 | 0.0 | 53.4 |
|  | No | n/a | Montana | Haven2_K1 | 18.5 | 0.0 | 0.0 | 61.7 |
|  | No | n/a | Montana | Haven2_K2 | 21.3 | 0.0 | 0.0 | 61.6 |
|  | No | n/a | Montana | Wild3_Y2X_R1 | 6.0 | 0.0 | 0.0 | 12.1 |
|  | No | n/a | Montana | Wild3_Y2X_R2 | 7.7 | 0.0 | 0.0 | 18.7 |
|  | No | n/a | Montana | Wild3_Y2X_R3 | 9.8 | 0.0 | 0.0 | 18.4 |
|  | No | n/a | Montana | Teton County | 20.2 | 0.0 | 0.0 | 31.4 |
|  | No | n/a | Montana | Charlis_32b_p2 | 9.4 | 0.0 | 0.0 | 15.5 |
|  | No | n/a | Montana | Charlis_40b_p4 | 21.9 | 0.0 | 0.0 | 27.2 |
|  | No | n/a | Montana | Billings | 14.5 | 0.0 | 0.0 | 25.7 |
|  | No | n/a | Montana | Billings | 12.4 | 0.0 | 0.0 | 24.3 |
|  | No | n/a | Montana | Billings | 14.1 | 0.0 | 0.0 | 25.4 |
|  | No | n/a | Montana | Billings | 19.2 | 0.0 | 0.0 | 35.3 |
|  | No | n/a | Montana | Billings | 14.7 | 0.0 | 0.0 | 15.3 |
|  | No | n/a | Montana | Billings | 16.3 | 0.0 | 0.0 | 26.0 |
|  | No | n/a | Montana | Billings | 17.2 | 0.0 | 0.0 | 32.8 |
|  | No | n/a | Montana | Billings | 16.0 | 0.0 | 0.0 | 24.9 |
|  | No | n/a | Montana | Billings | 13.7 | 0.0 | 0.0 | 22.4 |
|  | No | n/a | Montana | Billings | 15.5 | 0.0 | 0.0 | 25.4 |
|  | No | SBK-9 | Colorado | Milliken | 2.3 | 0.0 | 0.0 | 10.8 |
|  | No | SBK-9 | Colorado | Milliken | 2.2 | 0.0 | 0.0 | 13.4 |
|  | No | SBK-9 | Colorado | Milliken | 2.5 | 0.0 | 0.0 | 13.7 |
|  | No | SBK-9 | Colorado | Milliken | 2.5 | 0.0 | 0.0 | 13.8 |
| Haplotypes 1 and 2 |  |  |  |  |  |  |  |  |
|  | Yes | CO3R | Colorado | Cope | 2.9 | 0.0 | 0.0 | 9.9 |
|  | Yes | CO3R | Colorado | Cope | 3.3 | 0.0 | 0.0 | 11.3 |
|  | Yes | CO3R | Colorado | Cope | 2.2 | 0.0 | 0.0 | 15.4 |
|  | Yes | CO3R | Colorado | Cope | 5.4 | 5.8 | 1.7 | 16.2 |
|  | Yes | CO3R | Colorado | Cope | 2.5 | 0.0 | 0.0 | 16.4 |
|  | Yes | CO3R | Colorado | Cope | 2.2 | 0.0 | 0.0 | 17.7 |
|  | Yes | CO3R | Colorado | Cope | 3.0 | 2.4 | 1.7 | 19.0 |
| Haplotype 3 |  |  |  |  |  |  |  |  |
|  | Yes | ID1R | Idaho | Ada | 8.0 | 0.0 | 0.0 | 5.4 |
|  | Yes | ID1R | Idaho | Ada | 8.1 | 0.0 | 0.0 | 6.0 |
|  | Yes | OR1R | Oregon | Malheur | 4.3 | 0.0 | 0.0 | 7.0 |
|  | Yes | OR2R | Oregon | Malheur | 4.1 | 0.0 | 0.0 | 7.2 |
|  | Yes | OR3R | Oregon | Malheur | 3.6 | 0.0 | 0.0 | 7.0 |
|  | Yes | OR4R | Oregon | Malheur | 3.9 | 0.0 | 0.0 | 1.9 |
|  | Yes | OR5R | Oregon | Malheur | 2.3 | 0.0 | 0.0 | 2.9 |
|  | No | SBK-31 | Wyoming | Powell | 3.9 | 0.0 | 0.0 | 4.3 |
|  | No | SBK-31 | Wyoming | Powell | 5.7 | 0.0 | 0.0 | 7.2 |
|  | No | SBK-32 | Wyoming | Powell | 5.0 | 0.0 | 0.0 | 5.3 |
|  | No | SBK-32 | Wyoming | Powell | 4.4 | 0.0 | 0.0 | 4.6 |
| Haplotypes 1 and 3 |  |  |  |  |  |  |  |  |
|  | No | SBK-22 | Colorado | Eaton | 2.8 | 0.0 | 0.0 | 4.1 |
|  | No | SBK-22 | Colorado | Eaton | 4.5 | 0.0 | 0.0 | 10.4 |
|  | No | SBK-22 | Colorado | Eaton | 2.9 | 0.0 | 0.0 | 11.2 |
|  | No | SBK-22 | Colorado | Eaton | 3.9 | 6.0 | 2.1 | 15.7 |
|  | No | SBK-22 | Colorado | Eaton | 4.9 | 6.8 | 2.1 | 18.0 |
|  | No | SBK-22 | Colorado | Eaton | 3.3 | 6.4 | 2.2 | 25.2 |
|  | No | SBK-22 | Colorado | Eaton | 7.0 | 15.4 | 4.9 | 39.1 |
| Haplotypes 2 and 3 |  |  |  |  |  |  |  |  |
|  | Yes | WY1R | Wyoming | Powell | 5.2 | 0.0 | 0.0 | 4.8 |
|  | Yes | WY1R | Wyoming | Powell | 8.1 | 0.0 | 0.0 | 5.5 |
|  | Yes | WY1R | Wyoming | Powell | 7.2 | 0.0 | 0.0 | 12.9 |
|  | Yes | AB1R | Canada | Alberta | 10.6 | 0.0 | 0.0 | 6.3 |
|  | Yes | AB1R | Canada | Alberta | 11.0 | 0.0 | 0.0 | 7.5 |
|  | Yes | AB1R | Canada | Alberta | 3.7 | 0.0 | 0.0 | 10.9 |
|  | Yes | AB1R | Canada | Alberta | 3.7 | 0.0 | 0.0 | 10.9 |
|  | Yes | AB1R | Canada | Alberta | 6.3 | 0.0 | 0.0 | 21.2 |
|  | Yes | AB1R | Canada | Alberta | 6.3 | 0.0 | 0.0 | 21.2 |
|  | Yes | AB1R | Canada | Alberta | 6.6 | 0.0 | 0.0 | 24.0 |
|  | Yes | AB1R | Canada | Alberta | 6.6 | 0.0 | 0.0 | 24.0 |
|  | Yes | AB1R | Canada | Alberta | 6.8 | 0.0 | 0.0 | 24.9 |
|  | Yes | AB1R | Canada | Alberta | 6.8 | 0.0 | 0.0 | 24.9 |
|  | Yes | AB1R | Canada | Alberta | 6.6 | 0.0 | 0.0 | 25.9 |
|  | Yes | AB1R | Canada | Alberta | 6.6 | 0.0 | 0.0 | 25.9 |
|  | No | n/a | Montana | Denton | 1.9 | 0.0 | 0.0 | 4.0 |
|  | No | n/a | Montana | Denton | 7.8 | 0.0 | 0.0 | 17.8 |
|  | No | n/a | Montana | Denton | 4.9 | 0.0 | 0.0 | 21.1 |
|  | No | n/a | Montana | Cut Bank | 3.0 | 0.0 | 0.0 | 7.7 |
|  | No | n/a | Montana | Cut Bank | 5.4 | 0.0 | 0.0 | 16.2 |
|  | No | n/a | Montana | Carter | 11.1 | 0.0 | 0.0 | 6.0 |
|  | No | n/a | Montana | Carter | 6.2 | 0.0 | 0.0 | 20.3 |
|  | No | n/a | Montana | Carter | 1.7 | 0.0 | 0.0 | 4.0 |
|  | No | n/a | Montana | Carter | 1.8 | 0.0 | 0.0 | 12.6 |
|  | No | n/a | Montana | Carter | 3.2 | 0.0 | 0.0 | 13.4 |
|  | No | n/a | Montana | Carter | 9.4 | 0.0 | 0.0 | 12.4 |
|  | No | n/a | Montana | Carter | 8.6 | 0.0 | 0.0 | 12.6 |
|  | No | n/a | Montana | Carter | 2.5 | 0.0 | 0.0 | 1.0 |
|  | No | n/a | Montana | GIL 27 | 8.2 | 0.0 | 0.0 | 11.3 |
|  | No | n/a | Montana | GIL 28 | 12.9 | 0.0 | 0.0 | 19.0 |
|  | No | n/a | Montana | GIL 34 | 4.9 | 0.0 | 0.0 | 6.1 |
|  | No | n/a | Montana | GIL 35 | 15.0 | 0.0 | 0.0 | 21.2 |
|  | No | n/a | Montana | GIL 37 | 15.7 | 0.0 | 0.0 | 20.1 |
|  | No | n/a | Montana | GIL 38 | 4.3 | 0.0 | 0.0 | 6.1 |
|  | No | n/a | Montana | GIL 39 | 14.2 | 0.0 | 0.0 | 23.7 |
|  | No | n/a | Montana | GIL 44 | 13.4 | 0.0 | 0.0 | 19.2 |
|  | No | n/a | Montana | Vida | 14.2 | 0.0 | 0.0 | 7.7 |
|  | No | n/a | Montana | Vida | 18.4 | 0.0 | 0.0 | 9.2 |
| No EPSPS Gene Duplication | |  |  |  |  |  |  |  |
|  | Yes | KS4R | Kansas | Greeley | 0.5 | 0.0 | 0.0 | 2.6 |
|  | Yes | KS4R | Kansas | Greeley | 0.6 | 0.0 | 0.0 | 7.7 |
|  | Yes | KS4R | Kansas | Greeley | 0.5 | 0.0 | 0.0 | 8.4 |
|  | Yes | CO6R | Colorado | Otis | 1.2 | 0.0 | 0.0 | 5.8 |
|  | Yes | CO6R | Colorado | Otis | 1.0 | 0.0 | 0.0 | 6.4 |
|  | Yes | CO6R | Colorado | Otis | 1.1 | 0.0 | 0.0 | 6.9 |
|  | Yes | CO2R | Colorado | Brush | 1.0 | 0.0 | 0.0 | 1.2 |
|  | Yes | CO2R | Colorado | Brush | 0.7 | 0.0 | 0.0 | 2.8 |
|  | Yes | CO2R | Colorado | Brush | 0.8 | 0.0 | 0.0 | 6.5 |
|  | Yes | KS5S | Kansas | Meade | 1.0 | 0.0 | 0.0 | 3.1 |
|  | Yes | KS5S | Kansas | Meade | 1.2 | 0.0 | 0.0 | 3.4 |
|  | Yes | KS5S | Kansas | Meade | 1.1 | 0.0 | 0.0 | 3.9 |
|  | Yes | KS5S | Kansas | Meade | 1.1 | 0.0 | 0.0 | 4.7 |
|  | No | SBK-20 | Nebraska | Alliance | 0.9 | 0.0 | 0.0 | 11.0 |
|  | No | SBK-14 | Nebraska | Imperial | 0.7 | 0.0 | 0.0 | 3.6 |
|  | Yes | OK1R | Oklahoma | Cimarron | 0.9 | 0.0 | 0.0 | 3.4 |
|  | Yes | OK1R | Oklahoma | Cimarron | 1.0 | 0.0 | 0.0 | 5.1 |
|  | Yes | AB1R | Canada | Alberta | 0.6 | 0.0 | 0.0 | 2.1 |
|  | Yes | AB1R | Canada | Alberta | 0.6 | 0.0 | 0.0 | 2.1 |
|  | No | SBK-41 | Montana | Bighorn | 0.8 | 0.0 | 0.0 | 4.0 |
|  | No | SBK-41 | Montana | Bighorn | 0.6 | 0.0 | 0.0 | 5.1 |
|  | No | SBK-42 | Montana | Worden | 0.6 | 0.0 | 0.0 | 3.9 |
|  | No | SBK-42 | Montana | Worden | 0.7 | 0.0 | 0.0 | 5.7 |
|  | No | SBK-32 | Wyoming | Powell | 0.7 | 0.0 | 0.0 | 3.2 |
|  | No | SBK-31 | Wyoming | Powell | 0.9 | 0.0 | 0.0 | 4.3 |
|  | No | SBK-29 | Montana | Warren | 0.8 | 0.0 | 0.0 | 7.3 |
|  | No | SBK-29 | Montana | Warren | 0.9 | 0.0 | 0.0 | 12.4 |
|  | No | SBK-22 | Colorado | Eaton | 0.5 | 0.0 | 0.0 | 10.8 |
|  | No | SBK-22 | Colorado | Eaton | 0.6 | 0.0 | 0.0 | 3.2 |
|  | Yes | ID1R | Idaho | Ada | 1.4 | 0.0 | 0.0 | 4.1 |
|  | Yes | ID1R | Idaho | Ada | 1.2 | 0.0 | 0.0 | 4.1 |
|  | No | SBK-9 | Colorado | Milliken | 0.9 | 0.0 | 0.0 | 2.2 |
|  | No | SBK-9 | Colorado | Milliken | 0.7 | 0.0 | 0.0 | 7.7 |
|  | Yes | CO3R | Colorado | Cope | 0.8 | 0.0 | 0.0 | 6.8 |
|  | Yes | CO3R | Colorado | Cope | 1.2 | 0.0 | 0.0 | 11.0 |

Table S2. **Descriptive summaries of 11 microsatellite markers used to genotype 44 populations of kochia (*Bassia scoparia*).** Percent missing averaged across all individuals for each locus and then averaged across all loci, number of alleles observed at each locus across all individuals and averaged across loci, expected heterozygosity, calculated as Nei’s diversity index, and the distribution of alleles as evenness. Statistics were calculated for the entire dataset (All data) and then after locus/individuals were removed for missing data (Final data).

| Loci name | Percentage missing (%) | | Number of alleles | | H_e_ | | Evenness | |
| --- | --- | --- | --- | --- | --- | --- | --- | --- |
|  | All data | Final data | All data | Final data | All data | Final data | All data | Final data |
|  |  |  |  |  |  |  |  |  |
| SSR162 | 13.75 | NA | 4 | NA | 0.43 | NA | 0.71 | NA |
| SSR1225 | 1.77 | 0.79 | 4 | 4 | 0.37 | 0.37 | 0.56 | 0.56 |
| SSR1792 | 2.95 | 2.38 | 8 | 8 | 0.65 | 0.65 | 0.65 | 0.65 |
| SSR2656 | 2.95 | 2.88 | 7 | 7 | 0.44 | 0.44 | 0.61 | 0.61 |
| SSR2895 | 7.86 | 7.34 | 11 | 11 | 0.74 | 0.74 | 0.71 | 0.71 |
| SSR2916 | 6.68 | 6.75 | 6 | 6 | 0.58 | 0.58 | 0.82 | 0.82 |
| SSR5417 | 0.59 | 0.20 | 6 | 6 | 0.53 | 0.54 | 0.60 | 0.61 |
| SSR5608 | 1.77 | 1.79 | 5 | 5 | 0.53 | 0.53 | 0.71 | 0.71 |
| SSR5726 | 3.93 | 3.97 | 12 | 12 | 0.63 | 0.63 | 0.64 | 0.63 |
| SSR8376 | 0 | 0 | 4 | 4 | 0.45 | 0.45 | 0.81 | 0.82 |
| SSR3332 | 1.58 | 0.99 | 7 | 7 | 0.30 | 0.30 | 0.59 | 0.60 |
|  |  |  |  |  |  |  |  |  |
| Mean | 3.98 | 2.70 | 6.73 | 7 | 0.51 | 0.52 | 0.67 | 0.67 |

Table S3. **Descriptive summaries of 44 populations of *Bassia scoparia* genotyped at 11 SSR loci.** Number of individuals genotyped per population, percentage of missing data averaged across loci, number of alleles observed summed across all loci, percentage of the total alleles (n = 74) observed in each population averaged across loci, allelic richness averaged across all loci; H_O_, observed heterozygosity (H_o_), expected heterozygosity (H_e_), and F_IS_ and 95% confidence interval (CI).

| Population | Number  of individuals genotyped | Percentage missing (%) | Number of alleles observed | Percentage of total alleles observed (%) | Allelic richness | Ho | He | F_IS_ (95% CI) |
| --- | --- | --- | --- | --- | --- | --- | --- | --- |
|  |  |  |  |  |  |  |  |  |
| AB1R | 17 | 4.81 | 33 | 46.77 | 2.30 | 0.24 | 0.40 | 0.39 (0.24 – 0.53) |
| CO1R | 18 | 3.53 | 33 | 45.81 | 2.27 | 0.21 | 0.38 | 0.45 (0.35 – 0.55) |
| CO2R | 18 | 3.03 | 36 | 50.22 | 2.60 | 0.38 | 0.47 | 0.19 (0.06 – 0.32) |
| CO3R | 18 | 4.54 | 32 | 47.75 | 2.12 | 0.25 | 0.31 | 0.17 (0.00 – 0.33) |
| CO4R | 18 | 4.54 | 40 | 54.49 | 2.74 | 0.31 | 0.50 | 0.38 (0.25 – 0.50) |
| CO5R | 18 | 6.06 | 34 | 48.66 | 2.23 | 0.28 | 0.37 | 0.25 (0.07 – 0.40) |
| CO6R | 18 | 1.01 | 34 | 48.21 | 2.24 | 0.32 | 0.35 | 0.10 (-0.04 – 0.23) |
| CO7R | 18 | 1.51 | 40 | 57.03 | 2.74 | 0.41 | 0.48 | 0.13 (0.01 – 0.24) |
| CO8R | 18 | 2.52 | 34 | 49.25 | 2.42 | 0.41 | 0.45 | 0.10 (-0.02 – 0.23) |
| ID1R | 8 | 2.27 | 30 | 46.01 | 2.39 | 0.26 | 0.41 | 0.36 (0.09 – 0.55) |
| ID2R | 7 | 0 | 21 | 32.52 | 1.72 | 0.16 | 0.25 | 0.37 (-0.04 – 0.75) |
| KS10R | 9 | 0 | 31 | 41.16 | 2.46 | 0.43 | 0.44 | 0.02 (-0.24 – 0.23) |
| KS11R | 9 | 5.05 | 33 | 46.91 | 2.42 | 0.30 | 0.40 | 0.25 (0.10 – 0.35) |
| KS12R | 9 | 1.01 | 32 | 46.03 | 2.53 | 0.34 | 0.44 | 0.22 (0.06 – 0.33) |
| KS13R | 9 | 2.02 | 19 | 27.03 | 1.49 | 0.14 | 0.13 | -0.04 (-0.25 – 0.11) |
| KS14R | 9 | 3.03 | 32 | 43.55 | 2.38 | 0.25 | 0.38 | 0.35 (0.14 – 0.50) |
| KS15R | 9 | 6.06 | 35 | 48.56 | 2.64 | 0.34 | 0.46 | 0.27 (0.08 – 0.45) |
| KS1S | 9 | 2.02 | 34 | 50.38 | 2.6 | 0.41 | 0.5 | 0.17 (-0.06 – 0.34) |
| KS2S | 9 | 26.26 | 22 | 32.29 | 1.65 | 0.20 | 0.29 | 0.30 (-0.03 – 0.50) |
| KS3R | 9 | 0 | 30 | 42.69 | 2.39 | 0.28 | 0.47 | 0.39 (0.18 – 0.56) |
| KS4R | 9 | 9.09 | 25 | 35.39 | 2.04 | 0.31 | 0.37 | 0.15 (-0.13 – 0.37) |
| KS5S | 9 | 4.04 | 30 | 43.36 | 2.44 | 0.33 | 0.44 | 0.27 (0.06 – 0.42) |
| KS6S | 9 | 7.07 | 34 | 48.59 | 2.57 | 0.35 | 0.47 | 0.26 (0.05 – 0.44) |
| KS7S | 9 | 2.02 | 28 | 39.64 | 2.26 | 0.31 | 0.44 | 0.30 (0.01 – 0.51) |
| KS8S | 9 | 17.17 | 24 | 32.83 | 1.92 | 0.20 | 0.32 | 0.37 (0.06 – 0.66) |
| KS9R | 9 | 7.07 | 32 | 44.43 | 2.51 | 0.33 | 0.46 | 0.28 (0.04 – 0.47) |
| MT1R | 9 | 2.02 | 22 | 33.14 | 1.68 | 0.17 | 0.18 | 0.04 (-0.23 – 0.32) |
| MT2R | 9 | 5.05 | 28 | 40.45 | 2.21 | 0.34 | 0.40 | 0.14 (-0.03 – 0.25) |
| MT3R | 9 | 0 | 29 | 40.59 | 2.26 | 0.16 | 0.38 | 0.57 (0.29 – 0.82) |
| OK1R | 9 | 5.05 | 26 | 38.06 | 2.09 | 0.25 | 0.36 | 0.30 (0.12 – 0.46) |
| OR1R | 9 | 0 | 23 | 36.24 | 1.87 | 0.16 | 0.37 | 0.56 (0.21 – 0.82) |
| OR2R | 9 | 2.02 | 30 | 46.00 | 2.25 | 0.20 | 0.37 | 0.44 (0.26 – 0.58) |
| OR3R | 9 | 4.04 | 27 | 38.10 | 2.08 | 0.18 | 0.32 | 0.43 (0.21 – 0.58) |
| OR4R | 9 | 2.02 | 21 | 33.52 | 1.68 | 0.13 | 0.23 | 0.43 (0.05 – 0.66) |
| OR5R | 9 | 3.03 | 21 | 31.98 | 1.68 | 0.18 | 0.26 | 0.29 (0.06 – 0.45) |
| OR6R | 9 | 1.01 | 27 | 38.51 | 2.04 | 0.18 | 0.29 | 0.37 (0.17 – 0.51) |
| OR7R | 9 | 0 | 26 | 40.04 | 2.06 | 0.18 | 0.36 | 0.50 (0.24 – 0.74) |
| OR9S | 8 | 1.14 | 26 | 39.03 | 2.16 | 0.28 | 0.38 | 0.26 (0.03 – 0.42) |
| TX1R | 12 | 3.03 | 36 | 46.66 | 2.56 | 0.30 | 0.39 | 0.24 (0.07 – 0.43) |
| TX2R | 18 | 5.56 | 33 | 45.86 | 2.30 | 0.26 | 0.37 | 0.30 (0.13 – 0.43) |
| TX3R | 18 | 3.53 | 32 | 47.00 | 2.23 | 0.28 | 0.37 | 0.23 (0.09 – 0.35) |
| TX4R | 18 | 4.54 | 35 | 48.51 | 2.36 | 0.29 | 0.40 | 0.27 (0.12 – 0.40) |
| TX5R | 16 | 5.11 | 33 | 46.98 | 2.22 | 0.36 | 0.38 | 0.05 (-0.14 – 0.22) |
| WY1R | 9 | 3.03 | 33 | 44.24 | 2.58 | 0.33 | 0.47 | 0.31 (0.11 – 0.47) |
|  |  |  |  |  |  |  |  |  |


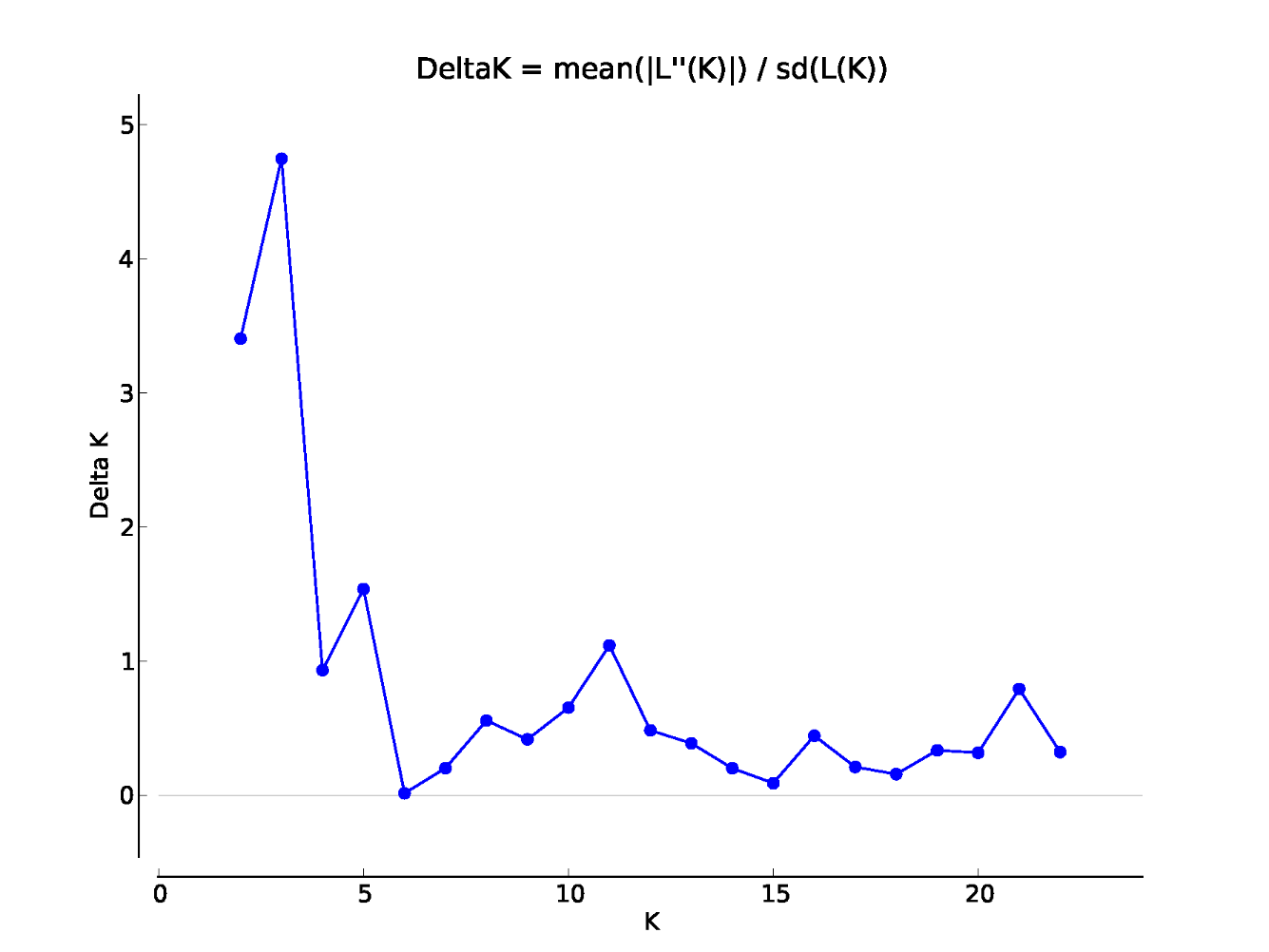


**Figure S1. Bayesian clustering analysis (STRUCTURE, Prichard et al., 2000) of kochia (*Bassia scoparia*) populations*.*** Analysis of the second order of change in ΔK from K=1-22 as a function of the number of clusters or gene pools, K, from the analysis of all samples supports K=3 as the most likely number of clusters or gene pools.

**Figure S2.** Principal components (PC) 1 and 2 and the percent of total variation explained from a principal component analysis of 504 individual kochia (*Bassia scoparia*) from 44 populations genotyped at 10 SSR loci. Individuals’ color and shape indicate their regional location (1 = Central Great Plains, 2 = Northern Plains, and 3 = Pacific Northwest). The inset displays all the eigenvalues in descending order.
